# The care of non-institutionalized ADL-dependent people in the Orcasitas neighborhood of Madrid (Spain) during the Covid-19 pandemic and its relationship with social inequalities, intergenerational dependency and survival

**DOI:** 10.3389/fpubh.2024.1411390

**Published:** 2024-09-25

**Authors:** Vicente Martín Moreno, María Inmaculada Martínez Sanz, Amanda Martín Fernández, Elena Sánchez Rodríguez, Irene Sánchez González, Julia Herranz Hernando, Miriam Fernández Gallardo, Miguel Recuero Vázquez, María Palma Benítez Calderón, Eva Sevillano Fuentes, Elena Pérez Rico, Laura Calderón Jiménez, Sara Guerra Maroto, Helena Alonso Samperiz, Irene León Saiz

**Affiliations:** ^1^Orcasitas Health Care Center, i+12 Research Institute of the Doce de Octubre Hospital, GIDO Collaborative Group Codirector, Madrid, Spain; ^2^Orcasitas Health Care Center, GIDO Collaborative Group Codirector, Madrid, Spain; ^3^Polibea Concert, GIDO Collaborative Group, Madrid, Spain; ^4^Orcasitas Health Care Center, GIDO Collaborative Group, Madrid, Spain; ^5^Orcasitas Health Care Center, GIDO Collaborative Group, Madrid, Spain

**Keywords:** activities of daily living, social inequalities, intergenerational dependency, gender inequalities, essential family caregiver, COVID-19, wheelchair, functional impairment

## Abstract

**Background:**

Mortality among people with dependency to perform basic activities of daily living (ADL) is higher than that of non-dependent people of the same age. Understanding the evolutionary course and factors involved in non-institutionalized ADL dependency, including the influence of the family structure that supports this population, would contribute to improved health planning.

**Methods:**

A longitudinal study carried out in the ADL-dependent population of the Orcasitas neighborhood, Madrid (Spain), between June 2020, when the nationwide COVID-19 lockdown ended, and June 2023. A total of 127 patients participated in the study, 78.7% of whom were women and 21.3% were men. Risk analysis was performed via odds ratios (OR) and hazard ratios (HR). Survival analysis was performed using Cox regression.

**Results:**

A total of 54.33% of the ADL-dependent persons did not live with their adult children and 45.67% did, being associated living independently with economic capacity and the married marital status but not with the dependency level. In women, being married increased the probability of living independently of their adult children (OR = 12.632; 95% CI = 3.312–48.178). Loss of mobility (OR = 0.398; 95% CI = 0.186–0.853), economic capacity of the dependent (HR = 0.596; 95% CI = 0.459–0.774), and living independently and having better economic capacity (HR = 0.471; 95% CI = 0.234–0.935) were associated with 3-year survival. Those who lived with their adult children had a worse autonomy profile and higher mortality (HR = 1.473; 95% CI = 1.072–2.024). Not being employed, not being married, and not owning a home were significantly associated with being an essential family caregiver. Caregivers were mostly women (OR = 1.794; 95% CI = 1.011–3.182).

**Conclusion:**

Among ADL-dependent persons, economic capacity influenced the ability to living independently and affected survival after 3 years. Loss of mobility (wheelchair use) was a predictor of mortality. Social inequalities promote that adult children end up as essential family caregivers. This generates reverse dependency and maintains a vulnerability that is transmitted from generation to generation, perpetuating social and gender inequalities. Dependent parent care in this cohort maintained an archaic pattern in which the eldest daughter cared for her parents. This study made it possible to show that ADL dependence is accompanied by complex interrelationships that must be considered in socio-health planning.

## Introduction

Elder care and health policies must be based on structural analysis of the populations to which they are directed, with the aim of maintaining equity and avoiding the appearance of social inequalities in their implementation (1–4). There are two clearly differentiated models of social and healthcare for people who are dependent on the performance of basic activities of daily living (ADLs). Living in the community or opting to live in a nursing home implies personal changes, but also different ways of managing dependency. Each model has advantages and disadvantages. Nursing homes provide permanent monitoring and care, which non-institutionalized persons lack. However, they also have vulnerabilities, which the COVID-19 pandemic highlighted. This pandemic had an unequal impact on these two groups of dependent people.

Functional ADL dependence is interrelated with vulnerability and frailty. Functional impairment has a socioeconomic impact at the personal, family, and community levels. Therefore, knowledge of the factors that contribute to vulnerability and frailty in the development of functional ADL dependence can enable preventive measures to be established early on (5).

The family structure plays a fundamental role in supporting non-institutionalized ADL-dependent persons, but the interrelationships between its members have rarely been studied. With respect to the traditional idea that children take care of their parents altruistically, it cannot be ruled out that other relationship models exist. These models could be masked by the presence of socioeconomically disadvantaged environments, or unfavorable personal situations (unemployment, divorce, difficulty of access to housing), which may be generating other models of cohabitation (6).

On the other hand, the presence in the media of older people taking care of their grandchildren while their adult children work, or taking them on vacation, creates a stereotypical image that retired people have disposable income. However, this media reality contrasts with the socioeconomic reality of a large part of this group. The level of economic income is a factor of both fragility and vulnerability (7). Socioeconomically disadvantaged environments have a lower life expectancy than the average of the cities that host them (6, 8). However, having fewer economic resources also limits access to a nursing home (9), or the hiring of home care assistants, negatively affecting the quality of life of these people.

In the current context, the COVID-19 pandemic has worsened the economic situation of people with less educational and/or professional training, who have higher unemployment rates and lower salaries. Salaries have been losing purchasing power due to inflation, increasing the number of people living at subsistence levels and poverty levels (8, 10).

This social situation has turned the pensions of our older people into an important source of income for some families. If not the only source of income (6, 8). This situation is favoring an increase in intergenerational inequalities (6). In 2021, almost one in three Spanish households was financially supported by a person over 65 years of age (11).

However, it is not known to what extent this situation is present in homes with dependent people over 65 years of age. Knowledge of these factors and early detection of other determinants that can cause and/or perpetuate social inequalities can allow health planning to be better adjusted to the real and perceived needs of the population (12). As a result, the management of the non-institutionalized ADL-dependent population will be better managed (13).

Related to health and socioeconomic determinants, life expectancy and healthy life expectancy are lower the lower the education level (14). This factor is affected by two situations. On the one hand, life expectancy is higher among women. On the other hand, women, due to previous social circumstances, have had fewer opportunities to study and enter the labor market. The result of this combination is that women in Spain currently receive a lower average pension than men. And this pension, since they have not worked, is generally a contributory widow’s pension, which represents a percentage of the pension received by their husband before his death. Or, if her husband is alive, a non-contributory pension, of a social nature for groups with no income, whose annual economic amount is below the level established for severe poverty (6). According to the Survey of Living Conditions in Spain published in 2021, 20.5% of people older than 64 years are at risk of poverty and/or social exclusion, and 5.8% suffer severe material and social deprivation (15). On the other hand, women are more frequently disabled. Furthermore, there is an unfavorable gender gap for women in relation to physical capacity and the prevalence of functional limitations to perform instrumental and basic activities of daily living (16).

Cross-referencing of data between the different public administrations (Ministry of Finance, Madrid Health Service) has made it possible to establish risk profiles and carry out actions that to some extent alleviate these shortcomings, such as free pharmacies for people with low incomes. However, much remains to be done in the field of social inequalities.

In the era of information and communication technologies, health systems have been equipped with computer applications that make it possible to establish specific situations in real time, both epidemiological and otherwise, and to make projections based on these data.

Specifically, in the Madrid Health System, the computer applications AP-Madrid, e-SOAP, Consulta-Web, Farma-Web, and EDO allow the permanent management of chronicity or the detection of real-time epidemiological outbreaks.

These applications provide global data by process, but also individual data, which specify the level of control and follow-up of each pathology, also allowing the detection of variations in the number of cases. These data, together with those provided by the City Councils, the Community of Madrid, and the Ministry of Finance, subsequently allow the preparation of the Atlas of Mortality and Social Inequalities of the Community of Madrid, which is prepared every 7 years; the Report on the State of Health of the Population of the Community of Madrid, which is prepared annually; and the Panel of Indicators of Districts and Neighborhoods of the Madrid City Council, which is prepared annually.

The accessibility of the data obtained through these applications has made it possible to know the impact of the COVID-19 pandemic. At the beginning of the COVID-19 pandemic, a new indicator called “COVID-19 Infection” was created in the AP-Madrid application, which allowed to register every day new cases, giving the Madrid Health System daily information on the evolution of the pandemic. This same process was created in all hospitals, so that the daily hospital admissions were also known. There were also deaths. This new indicator was also incorporated into the IT-Web application, a module that records the reasons for sick leave. This made it possible to know not only the number of people who took time off work each day due to the COVID-19 infection, but also the number of people who, according to the regulations established by the Spanish Government, took time off work due to illnesses that entailed a risk of mortality if they contracted COVID-19.

However, much work remains to be done, not only in relation to its impact on certain groups, but also in relation to what we must learn to face future crises.

In relation to ADL-dependent persons and in the context of the COVID-19 pandemic, the initial global view, based on what happened in nursing homes, showed high mortality and functional deterioration (17). In Spain, approximately 3% of cases and 40% of deaths have occurred in nursing homes. The ease of studying closed clusters, such as nursing homes, as opposed to carrying out these studies during confinement in ADL-dependent persons living in their own homes, meant that much evidence was generated on what happened in these centers, and little on what happened in the homes. Conducting a home-by-home study, during confinement due to a pandemic that was causing high mortality, meant exposing dependents and the professionals themselves to risk. However, knowing the response to this new stressor of frail people living in their environment was a necessity, with obvious usefulness in health planning.

Within this planning, it is usually assumed that people with ADL dependency are being cared for by others, but analyzing to what extent this premise corresponds to reality requires specific studies. Population aging generates an increasing number of people with functional limitations. It is currently the baby-boom generation that is facing this situation, apparently presenting adequate potential to cope with the care of their parents as they age. However, social and work circumstances have also changed. The expectations of life of this generation are not those of their parents. Furthermore, studies are needed to analyze the social changes that are taking place. Studies whose complexity must go beyond establishing this relationship and its characteristics, also addressing other factors, such as economic and social factors. Among the social factors, the epidemic of loneliness that affects many people, including the older adult, probably also plays a role in this network that ends up shaping the models of care for our older people.

On the other hand, the population aging process runs parallel to the decline in the birth rate, and both factors will affect the care of the older persons in the next few decades. The established social model, where children take care of their parents when the latter need it because of their advanced age or the presence of functional limitations, will probably cease to be sustainable. However, the impact of this phenomenon in the short, medium, and long term has not been clearly established. This situation justifies studies that analyze the current situation, and particularly the situation of the most vulnerable population, with the aim of developing strategies through social and health planning that maintain equity and promote socially healthy aging.

To ensure that as many people as possible benefit from healthy aging, it is necessary to analyze the factors involved in this process. Among them is the management of care for the older people. To foresee in aging is likely to increase the quality of life and survival, as well as allow the detection of vulnerable groups, not only among the older adult, but also among the caregivers. Because, within the people who provide socio-family support to the dependent person, pockets of poverty and intergenerational dependency could be masked.

Based on these premises, we proposed to carry out the present study, taking as our reference population the population over 65 years of age in the Orcasitas neighborhood of Madrid (Spain). Within this population group, the target population was the population functionally dependent on the performance of basic activities of daily living that was not institutionalized. It was proposed to carry out the study at the end of the confinement in the usual home due to the COVID-19 pandemic. The initial working hypothesis was that there would have been a deterioration in the baseline conditions because of the confinement and the social isolation measures imposed to face the pandemic. The objectives were: (1) To analyze the figure of the person with functional dependency. (2) To carry out a descriptive analysis of the family structure that supports people with functional ADL dependency. (3) To analyze the figure of the essential family caregiver. (4) To analyze specifically whether there is economic or housing interdependence between the people who live in the dependent person’s home and the dependent person himself or herself. (5) In parallel, we will analyze the demographic, socioeconomic, and cultural factors that they share and the association with the availability of socio-health resources, such as public or private home assistants or internal caregivers. (6) Analyze the interrelationships between these factors and functional ADL dependence. (7) To analyze the interrelationships between all these factors and survival at 3 years.

## Materials and methods

### Design and study population

A longitudinal descriptive study was carried out in the non-institutionalized functionally ADL-dependent population of the Orcasitas neighborhood of Madrid (Spain) between June 2020 and June 2023. This cohort was denominated the Orcasitas cohort. Inclusion criteria were to be 65 years of age or older and to be included in the dependency protocol of the e-SOAP application of the Madrid Health Service for the Orcasitas health center. Exclusion criteria included not belonging to the basic health area, being hospitalized or displaced to another home, or having a diagnosis of terminal illness. Fourteen patients were excluded. Five patients refused to participate in the study, and four had died between the time the e-SOAP database was obtained and the start of the study. In the end, 127 patients participated.

Within the protocol for the care of the older persons contained in the Primary Care Services Portfolio, the entire population over 70 years of age should be screened for functional dependence by means of the Barthel index. This screening is also carried out for people between 65 and 70 years of age who have been assigned a high level of intervention due to their comorbidities. And in those persons in whom, from the medical or nursing consultation, functional limitations are detected in the examination.

### Data collection

A previously validated questionnaire was used to obtain the variables to be studied. These variables were: (1) age, (2) sex, (3) marital status, (4) educational level, (5) income level, (6) family structure (with whom she lives; number of children; how many still live in the family home and their gender, marital status of cohabiting adult children, employment status of cohabiting adult children and whether these cohabiting adult children had their own home; the presence of other residents and their gender were also analyzed), and (7) having a public benefit of a housework assistant at home by social services (PSHA), private housework assistant (PHA), or live-in caregiver. We also recorded the number of chronic diseases, the number of active principles consumed, and whether these persons had private health insurance.

The level of functional dependence on the performance of basic activities of daily living was assessed using the Barthel index. Following the recommendations of the health system of the community of Madrid, any person with a Barthel index score of 60 or less was considered dependent. Within the population recognized as functionally dependent, the recommendations of our health system included two categories, establishing a cutoff point of 40 on the Barthel index: moderate dependence (Barthel 40–60) and severe dependence (Barthel less than 40). The “moderate dependence” category included the categories of the classic Barthel index classification “mild dependence” (Barthel 60) and “moderate dependence” (Barthel 40–55). The “severe dependence” category included the categories of the classic Barthel index classification “severe dependence” (Barthel 20–35) and “total dependence” (Barthel less than 20).

The use of mobility assistance devices implies an alteration of autonomy of greater or lesser intensity, which can lead dependent persons to make decisions about their way of life in the community or to decide to be institutionalized. To assess the influence of this parameter on the way dependent persons in this cohort live together, the use of wheelchairs, walkers, or crutches was analyzed as possible determinants of the type of family structure.

For the assessment of economic factors, the information provided by the Individual Health Card (IHC) platform of the Health System of the Community of Madrid was used. This platform establishes two categories of economic income: (1) above 11,200 euros/year and (2) less than 11,200 euros/year. This economic threshold corresponds to the level of co-payment of pharmaceutical care. Users with an income of less than 11,200 euros/year are exempt from paying the cost of the drugs they receive. Consequently, this threshold of 11,200 euros/year can be used as an indicator of a person with low financial resources. The TSI platform obtains these data from the Ministry of Finance.

With respect to marital status, the multiplicity of situations that can occur in social reality recommends using only two categories for data analysis: (1) married, which includes being married, having a common-law partner, or living habitually with another person in a non-marital affective relationship, regardless of their gender; and (2) unmarried, which includes all other marital status situations (single, widowed, separated, divorced, etc.). The real situation of each person was recorded in the database.

Operationally, the following definitions were used: (1) Cohabiting children: a child who resided at the same address as the dependent person. (2) First child: older child who lived with his dependent family member and participated in his care, although there could also be cohabiting younger children who shared this activity and were even the main caregivers of the dependent person. (3) Essential family caregiver (EFC): only child who resided in the same home as the dependent person and provided the care needed in an efficient and nondelegated manner. Or, when several children lived together, the child assumed the principal responsibility of providing the necessary care to the dependent person.

The type of residential solution that the functionally dependent persons adopted to reside in the community was defined as “housing situation.” The housing situation of functionally dependent persons was classified into three groups: (1) living independently; (2) living with their children; and (3) living with other people. To analyze the data, this criterion was used to define the actual housing situation, but the data were also grouped into two groups with the following criterion: (1) they live with their children or with other people and (2) they live independently.

Intergenerational dependence was analyzed in accordance with the following items: (1) having or not having a house of one’s own; (2) having or not having a work; and (3) regardless of marital status, continuing to live with parents beyond the age of 35.

Interdependence required two situations. The presence of adult children essential family caregivers who did not have economic-social autonomy because they did not have their own home, job, and/or were living alone or with their family in the home of a dependent person. A dependent person whose degree of dependency requires care by other people due to their level of dependency, loneliness, low economic resources, marital status, and/or need for a wheelchair.

A cutoff point of five was established for the analysis of the variable number of chronic diseases, with two groups: (1) they have less than five chronic diseases; (2) they have five or more chronic diseases.

For the analysis of the drug prescription variable, a cutoff point of five was established, with two groups: (1) they have less than five active principles prescribed; (2) they are prescribed five or more active principles.

Three reference points were used for the analysis of the age variable: (1) chronological age; (2) age with a cutoff point of 80 years and two population groups, under 80 years and with age equal to or over 80 years; and (3) age with a cutoff point of 90 years and two population groups, younger than 90 years and with age equal to or older than 90 years.

In Spain, the level of income of retired senior citizens is updated each year in accordance with the Consumer Price Index (CPI). As a result, the pensions received by the older adult maintain their purchasing power at a level like the increase in the cost of living each year, with this variable acting as a constant for the economic level within each economic income level group.

### Data analysis

The variables recorded were analyzed with SPSS 18.0, and the normality of the data was assessed with the Shapiro–Wilk test. When the outcome variable did not follow a normal distribution, ANOVA was performed with Levene’s homogeneity test, applying Welch’s correction as a robust test of equality of means when the sample size is less than 30. Differences between continuous variables were analyzed using the Student’s *t*-test, the Mann–Whitney *U* test, or the Kruskal–Wallis test, and differences between categorical variables were analyzed using the chi-square test. The probability of occurrence of an event was analyzed by odds ratio (OR).

We followed the next steps: Univariate analysis was performed to identify variables associated with long-term survival of individuals included in this cohort. Covariates that were significant in the bivariate analysis were included in the survival analysis using Cox regression. Cox regression was used, with a proportional hazard’s survival regression model. This model assumes that the effect of the predictor variables remains constant over time. The principal comparison variable with survival was the housing situation (living independently, living with children, living with others) of the ADL-dependent persons. The resulting model was summarized using the estimated coefficients, *p*-values, and hazard ratios (HR), with their 95% confidence intervals. *Post-hoc* tests were performed by linear regression analysis and a stepwise model, also using the Wald test and Bonferroni correction for multiple comparisons. Any *p* < 0.05 was considered significant.

### Ethics statement

This study was approved by the Local Research Commission of the Assistance Directorate Center dependent on the Primary Care Management of the Department of Health of the Community of Madrid (Spain), resolution 16/20-C-Bis, of 29 June 2020. The Ethics Committee of Hospital Universitario Doce de Octubre endorsed this as sufficient approval, resolution 23/501 of 26 September 2023.

## Results

### Demographic characteristics

The mean age of the 127 ADL-dependent persons in the Orcasitas cohort was 86.6 years, with a predominance of women (78.7%), who were mostly widowed (71%), over men, who were generally married (55.6%). Although the number of children ranged between 0 and 12, two to four children were the norm (3.11 ± 1.827), for a total of 395 children. Of these, one in five continued to live with their parents, with up to four children currently living at the same address. The demographic and family structure data are shown in Tables 1–3. In this cohort, 3.1% (4) of the members of this cohort had private health insurance.

**Table 1:** Sociodemographic and socioeconomic data of the functionally dependent population of Orcasitas (Orcasitas cohort).

Variable	Value	Variable	Value
Age (mean ± SD)	86 ± 6.304	BMI	28.58 ± 4.701
Sex:		Live with a partner:	
Male	27 (21.3%)	Yes	39 (30.7%)
Female	100 (78.7%)	No	86 (67.7%)
Income level^a^:		Salary and Barthel score (mean ± SD)^b^:	
<11,200 euros/year	60 (47.2%)	<11,200 euros/year	39.26 ± 19.13
≥11.200 euros/year	67 (52.8%)	≥11,200 euros/year	47.00 ± 17.95
Marital status:		Studies:	
Married	39 (30.7%)	Insufficient	111 (87.4%)
Widower	82 (64.6%)	Primary	10 (7.9%)
Separated	3 (2.4%)	Media	4 (3.1%)
Single	3 (2.4%)	Superiors	1 (0.8%)
Number of children (*n* = 395)^a^		Number of children (*n* = 395)	
0	4 (3.1%)^a^	5	5 (3.9%)
1	14 (11%)	6	8 (6.3%)
2	32 (25.2%)	8	2 (1.6%)
3	34 (26.8%)	10	1 (0.8%)
4	26 (20.5%)	12	1 (0.8%)
Live with children/marital status:		Does not live with children/marital status:	
Married	10 (17.2%)	Married	29 (42%)
Separated/divorced/widowed/single	48 (82.8%)	Separated/divorced/widowed/single	40 (58%)
Live with children/income level:		Does not live with children/ income level:	
<11.200 euros/year	33 (56.9%)	<11,200 euros/year	27 (39.1%)
≥11.200 euros/year	25 (43.1%)	≥11,200 euros/year	42 (60.97%)
Public assistant at home:		Private home assistant:	
Yes	70 (55.1%)	Yes	45 (35.4%)
No	56 (44.1%)	No	79 (62.2%)
No answer	1 (0.8%)	No answer	3 (2.4%)
Attendant loss due to confinement	44 (62.8%)	Attendant loss due to confinement	10 (22.2%)
Live-in assistant:		Barthel level:	
Yes	24 (18.9%)	Severe (under 40)	38 (29.9%)
No	101 (79.5%)	Moderate (40–60)	89 (70.1%)
No answer	2 (1.6%)	Caregiver of your partner:	
Attendant loss due to confinement	0 (0%)	Yes	7 (5.5%)
Assistant confined with patient	4 (16.7%)	No	109 (85.8%)

a Information provided by the Individual Health Card (IHC) platform of the Health System of the Community of Madrid.

b Mean score in the Barthel index according to having a financial income of more or less than 11,200 euros/year. Percentages over *n* = 127.

**Table 2:** Family structure of people with functional dependence in Orcasitas (Orcasitas cohort).

Variable	Cases	Variable	Cases
Type of cohabitant (*n* = 269)		Sex of first son caregiver^a^	
Children	77 (28.6%)	Male	19 (33.3%)
Other people	192 (71.4%)	Woman	38 (66.7%)
Residents by address^b^ (*n* = 269)		Female residents (*n* = 170)	
0	11 (8.7%)	0	21 (16.5%)
1	26 (20.5%)	1	55 (43.3%)
2	51 (40.2%)	2	43 (33.9%)
3	24 (18.9%)	3	5 (3.9%)
4	8 (6.3%)	4	1 (0.8%)
5	5 (3.9%)	5	2 (1.6%)
6	2 (1.6%)		
Male residents (*n* = 99)		Number of cohabiting children (*n* = 77)	
0	51 (40.2%)	1	47 (61%)
1	60 (47.2%)	2	7 (18.2%)
2	10 (7.9%)	3	4 (15.6%)
3	5 (3.9%)	4	1 (5.2%)
4	1 (0.8%)		
Female cohabiting children (*n* = 45)		Male cohabiting children (*n* = 32)	
1	33 (73.3%)	1	27 (84.4%)
2	3 (13.3%)	2	1 (6.2%)
3	2 (13.3%)	3	1 (9.4%)
Cohabiting children (*n* = 77)		Cohabiting children age (mean ± SD):	
First child	57 (74%)	First child	57.56 ± 8.46
Second child	14 (18.2%)	Second child	53.21 ± 8.31
Third child	5 (6.5%)	Third child	55.40 ± 8.44
Fourth child	1 (1.3%)	Fourth child	41.00 *n* = 1
Marital status cohabiting child (*n* = 77)		First child^a^ marital status (*n* = 57)	
Married	22 (28.95%)	Married	16 (28.1%)
Widower	3 (3.95%)	Widower	2 (3.5%)
Separated/divorced	17 (22.37%)	Separated/divorced	12 (21%)
Single	36 (47.37%)	Single	27 (47.4%)
Cohabiting children works (*n* = 77)		First child^a^ works (*n* = 57)	
Yes	37 (48%)	Yes	25 (43.9%)
No	39 (50.7%)	No	31 (54.4%)
Do not know	1 (1.3%)	It does not consist	1 (1.7%)
Cohabiting children with own house (*n* = 77)		First child^a^ own house (*n* = 57)	
Yes	29 (37.7%)	Yes	17 (29.8%)
No	48 (62.3%)	No	40 (70.2%)
Cohabiting children age cutoff point:		First child^a^ age cutoff point:	
Over 55 years old	38 (48.7%)	Over 55 years old	35 (61.4%)
Under 55 years old	40 (51.3%)	Under 55 years old	22 (38.6%)

a First child: the oldest son of the family who lives with their dependent relative and takes care of them, although there may be more cohabiting adult children who share this activity and are even the main caregivers of the dependent person.

b Number of people living in the household of the dependent other than the dependent.

**Table 3:** Independence within the dependency: effects of sociodemographic, economic, and sociosanitary variables in relation to the social situation of the dependent person living with the adult children or independent of them.

Autonomy, dependence, and interdependence I			
Functional dependence: living independently while being dependent			
Variable	Value *n* (%)	Statistic value	Significance value
Residential situation of dependents:			
Live with their adult children	57 (44.90%)	–	–
Do not live with their adult children	70 (55.10%)		
Live with a adult children and are male	14 (11.02%)	ꭕ^2^ = 0.528	*p* = 0.467
Live with a adult children and are female	44 (34.65%)		
Do not live with adult children and are male	13 (10.24%)		
Do not live with adult children and are female	56 (44.09%)		
Live with adult children, income ≥11,200 euros/year	25 (19.69%)	ꭕ^2^ = 3.991OR^a^ = 2.053	*p* = 0.046CI^b^ = 1.010–4.176
Live with adult children, income <11,200 euros/year	33 (25.98%)		
Do not live with adult children, income ≥11,200 euros/year	42 (33.07%)		
Do not live with adult children, income <11,200 euros/year	27 (21.26%)		
Live with adult children and are married	10 (7.87%)	ꭕ^2^ = 9.099OR = 0.287	*p* = 0.00395% CI = 0.125–0.660
Live with adult children and are unmarried	48 (37.80%)		
Do not live with adult children and are married	29 (22.83%)		
Do not live with adult children and are unmarried	40 (31.50%)		
Live with adult children and Barthel under 40	20 (15.75%)	ꭕ^2^ = 1.134	*p* = 0.769
Live with adult children and Barthel 40 or higher	38 (29.92%)		
Do not live with adult children and Barthel under 40	18 (14.17%)		
Do not live with adult children and Barthel 40 or higher	51 (40.16%)		
Live with adult children and Barthel score (mean ± SD)	41.47 ± 20.02	z = −0.745	*p* = 0.456
Do not live with adult children and Barthel score (mean ± SD)	44.80 ± 18.14		
Live with adult children and are at high risk	32 (25.20%)	ꭕ^2^ = 0.439	*p* = 0.508
Live with adult children and a medium or low risk	26 (20.47%)		
Do not live with adult children and are at high risk	34 (26.77%)		
Do not live with adult children and a medium or low risk	35 (27.56%)		
Live with adult children and need a high intervention level.	24 (18.90%)	ꭕ^2^ = 0.181	*p* = 0.671
Live with adult children and need a medium-low intervention level.	34 (26.77%)		
Do not live with adult children and need a high intervention level.	26 (20.47%)		
Do not live with adult children and need a medium-low intervention level	43 (33.86%)		

a OR, odds ratio.

b CI, 95% confidence interval.

### ADL-dependent population groups depending on the type of living together at home

Depending on the type of residential solution that the functionally dependent persons in this cohort adopted for living in the community, three main groups of cohabitation modes were observed: dependents living alone, dependents living with their partner, and dependents living with other persons (Table 4).

**Table 4:** Socio-sanitary and economic factors related to the care of the dependent population.

Socio-sanitary and economic factors in relation to the care of the dependent population					
Variable/population groups	Group 1^a^ (*n* = 25)	Group 2^b^ (*n* = 22)	Group 3a^c^ (*n* = 11)	Group 3b^d^ (*n* = 47)	Group 3c^e^ (*n* = 16)
Functional disability:					
Severe	8 (33.3%)	3 (13%)	2 (20%)	18 (38.3%)	6 (33.3%)
Moderate	16 (66.7%)	20 (87%)	8 (80%)	29 (61.7%)	12 (66.7%)
Wheelchair:					
Yes	5 (20.8%)	5 (21.7%)	4 (40%)	17 (36.2%)	9 (50%)
No	19 (79.2%)	18 (78.3%)	6 (60%)	30 (63.8%)	9 (50%)
Walker:					
Yes	6 (25%)	8 (34.8%)	4 (40%)	20 (42.6%)	7 (38.9%)
No	18 (75%)	15 (65.2)	6 (60%)	27 (57.4%)	11 (61.1%)
Crutches/Cane:					
Si	10 (41.7%)	13 (56.5%)	5 (50%)	20 (42.6%)	3 (16.7%)
No	14 (58.3%)	10 (43.5%)	5 (50%)	27 (57.4%)	15 (83.3%)
PSHA^f^:					
Yes	16 (66.7%)	20 (87%)	2 (20%)	22 (47.8%)	6 (33.3%)
No	8 (33.3%)	3 (13%)	8 (80%)	24 (52.2%)	12 (66.7%)
PHA^g^:					
Yes	5 (21.7%)	4 (18.2%)	2 (20%)	14 (30.4%)	15 (83.3%)
No	18 (78.3%)	18 (81.8%)	8 (80%)	32 (69.6%)	3 (16.7%)
Internal caregiver:					
Yes	0	1 (4.3%)	0	7 (15.2%)	12 (70.6%)
No	24 (100%)	22 (95.7%)	10 (100%)	39 (84.8%)	5 (29.4%)
Income level:					
>11,200 euros/year	10 (41.7%)	8 (34.8%)	3 (30%)	30 (63.8%)	8 (44.4%)
<11,200 euros/year	14 (58.3%)	15 (65.2%)	7 (70%)	17 (36.2%)	10 (55.6%)
Survival June 2023:					
Alive	16 (66.7%)	18 (78.3%)	6 (60%)	22 (46.8%)	10 (55.6%)
Deceased	8 (33.3%)	5 (21.7%)	4 (40%)	25 (53.2%)	8 (44.4%)

Dependent population groups depending on the type of living together at home.

a Dependent individuals who lived alone.

b Dependent persons who lived with their partner.

c Married dependent persons who lived with their adult children.

d Unmarried dependents who lived with their adult children.

e Unmarried dependents who lived with other people who were not adult children.

f Assistant assigned to them for housework by social services.

g Private housework assistant.

Within the first group, which was made up of 18.9% (*n* = 24) of ADL dependents living alone, 33% had severe functional disability. For mobility at home or away from home, 21% required a wheelchair, 25% needed a walker, and 42% needed crutches/cane. As external support and in relation to their care needs, 67% had a PSHA assistant, 22% had an hourly PHA assistant, and no ADL-dependent person in this group had an internal caregiver. Fifty-eight percent had an income greater than 11,200 euros/year. Survival at 3 years was 67%, with 33% dying during this period (Table 4, group 1).

In the second group, 18.1% (*n* = 23) of the ADL-dependent persons lived with their partner, and 13% had severe functional disability. When displacing inside or outside the home, 22% used a wheelchair, 35% used a walker, and 56.5% used crutches/cane as a means of support. Their personal situation meant that 87% needed a PSHA assistant, 18.2% a PHA assistant, and 4.3% an internal caregiver. The partner was the primary caregiver for 20.5% of these dependents, and 5.5% of these dependents were the primary caregiver for their dependent partner. Sixty-five percent had an income greater than 11,200 euros/year. Survival at 3 years in this group was 78%, with 22% of its members dying during this period (Table 4, group 2).

Finally, a third group, comprising 63% (*n* = 80) of the persons in this cohort, lived with other persons. This cohabitation with other people could be carried out with their partner (18.75%, *n* = 15), or individually (81.25%, *n* = 65), when they were widowed, single, or separated/divorced dependents.

In this third group, several subgroups were observed.

The first subgroup (8%; *n* = 10) was married dependents living with their children. In this subgroup, 20% had a severe functional disability. As support devices for mobility inside or outside the home, 40% used a wheelchair, 40% used a walker, and 50% used crutches/cane. Because of their functional limitations, 20% of these individuals had a PSHA assistant and 20% had a PHA assistant, while none had an in-house caregiver hired. Seventy percent had an income greater than 11,200 euros/year. At 3 years, 60% still lived and 40% had died (Table 4, group 3a). Another 4% (*n* = 5) of married dependents lived with other people who were not adult children (nephews, grandchildren, brothers).

The second subgroup (37%; *n* = 47) were unmarried ADL dependents who lived with their children. Thirty-eight percent had a severe functional disability. As mobility aids, 36% used a wheelchair, 43% used a walker, and 43% used crutches. In terms of support for care and housework, 48% had a PSHA assistant, 30% had a PHA assistant, and 15% had an internal caregiver. A total of 36% had an income greater than 11,200 euros/year, and 85% (40) were women. Survival in this subgroup at 3 years was 47% (Table 4, group 3b).

Finally, a third subgroup, representing 14% (*n* = 18), were unmarried dependents who lived with other people who were not adult children (Table 4). Among the people in this group, 33% had severe functional dependence. In this subgroup, there was a higher percentage of dependent persons who used a wheelchair to move around the home or outside the home (50%). As for other mobility aids, 39% used a walker and 17% used crutches/cane. This subgroup had the lowest percentage of people using crutches/cane. As regards supports for daily tasks at home and for personal care, 33% had a PPATD assistant, 83% had an APTD, and 71% had an internal caregiver. In this subgroup, the highest percentage of private caregivers (APTD) and internal caregivers was observed with respect to the Orcasitas cohort. Forty-four percent had an income greater than 11,200 euros/year. Survival in this group at 3 years was 56% (Table 4, group 3c).

### Typology of people who live with patients with functional dependence and the role of essential family caregiver

This heterogeneous third group lived with a total of 269 people, 63.2% of whom were women and 36.8% were men, with an average occupancy per dwelling of three people, not counting dependents.

Regarding kinship, 28.25% (*n* = 77) were children, representing 19.49% of the total number of children. It was common to live with only one child (60.5%), generally, the first child (74%), who had an average age of 56.74 years and was more frequently a woman (67.7%) than a man (33.3%). These adult children had a social situation that showed that 47.37% were single, another 21.05% were separated (Table 2), 54.39% did not work, and 70.18% did not own their own home (Table 5).

**Table 5:** Dependence and survival.

Variable	Value *n* (%)	Statistic value	Significance value
Level of economic income:			
**Alive in 2023**			
Income over 11,200 euros/year	47 (37.01%)	ꭕ^2^ = 7.219OR^b^ = 0.372	*p* = 0.007CI^c^ = 0.180–0.772
income less than 11,200 euros/year	28 (22.05%)		
**Deceased in 2023**			
income over 11,200 euros/year	32 (25.19%)		
Income less than 11,200 euros/year	20 (15.75%)		
Residential situation of the dependent person:			
**Alive in 2023**			
Live with adult children	29 (22.84%)	ꭕ^2^ = 3.620	*p* = 0.057
Does not live with adult children	46 (36.22%)		
**Deceased in 2023**			
Live with adult children Does not live with adult children	29 (22.83%)23 (18.11%)		
Caregiving adult children worksCaregiving adult children does not work	25 (43.86%)32 (56.14%)	ꭕ^2^ = 0.072	*p* = 0.788
Caregiving adult children works adjusted by dependent’s income	–	HR^d^ = 1.070	95% CI = 0.644–1.777
Married caregiving adult childrenUnmarried caregiving adult children (single, widowed, and separated/divorced)	17 (29.82%)40 (70.18%)	ꭕ^2^ = 0.141	*p* = 0.707
Unmarried caregiving adult children adjusted for dependent’s income	–	HR = 0.969	95% CI = 0.540–1.741
Caregiving adult children who own the house where the dependent residesNon-homeowner caregiving adult children	17 (29.82%)40 (70.18%)	ꭕ^2^ = 0.912	*p* = 0.340
Non-homeowner caregiving adult children, adjusted for dependent person’s income level	–	HR = 0.788	95% CI = 0.419–1.485
Age of cohabiting caregiving adult children^a^:			
55 or more years	35 (61.40%)	ꭕ^2^ = 3.020	*p* = 0.082
<55	22 (38.60%)		
Number of cohabiting adult children:			
1	44 (77.19%)	ꭕ^2^ = 5.208OR = 4.815	*p* = 0.02295% CI = 1.160–19.985
2 or more children	13 (22.81%)		
Residential situation of dependent people (*n* = 104):			
They live with their adult children	57 (54.81%)	ꭕ2 = 5.768OR = 2.709	*p* = 0.01695% CI = 1.189–6.172
They live alone or with their partner	47 (45.19%)		
PSHA (*n* = 126)^e^:			
Lives independently and has PSHA	36 (28.57%)	ꭕ^2^ = 13.442	*p* = 0.001
Lives independently and does not have PSHA	11 (8.73%)		
Lives with adult children and has PSHA	24 (19.05%)		
They live with adult children and do not have PSHA	32 (25.40%)		
Lives with other people and has PSHA	10 (7.94%)		
They live with other people and do not have PSHA	13 (10.31%)		
PHA (*n* = 124)^f^:			
Lives independently and has PHA	9 (7.26%)	ꭕ^2^ = 32.145	*p* < 0.001
Lives independently and does not have PHA	36 (29.03%)		
Lives with adult children and has PHA	16 (12.90%)		
They live with adult children and do not have PHA	40 (32.26%)		
Lives with other people and has PHA	20 (16.13%)		
They live with other people and do not have PHA	3 (2.42%)		
LIC (*n* = 125)^g^:			
Lives independently and has LIC	1 (0.80%)	ꭕ^2^ = 51.082	*p* < 0.001
Lives independently and does not have LIC	46 (36.80%)		
Lives with adult children and has LIC	7 (5.60%)		
They live with adult children and do not have LIC	49 (39.20%)		
Lives with other people and has LIC	16 (12.80%)		
They live with other people and do not have LIC	6 (4.80%)		

Effects of different sociodemographic variables on survival at 3 years.

a The cutoff point for the age of the cohabiting caregiver was set at 55 years, the age of early retirement in Spain.

b OR, odds ratio.

c CI, 95% confidence interval.

d HR, Hazard ratio.

e PSHA, assistant assigned to them for housework by social services.

f PHA, private homework assistant.

g LIC, live-in caregiver.

**Figure 1:**
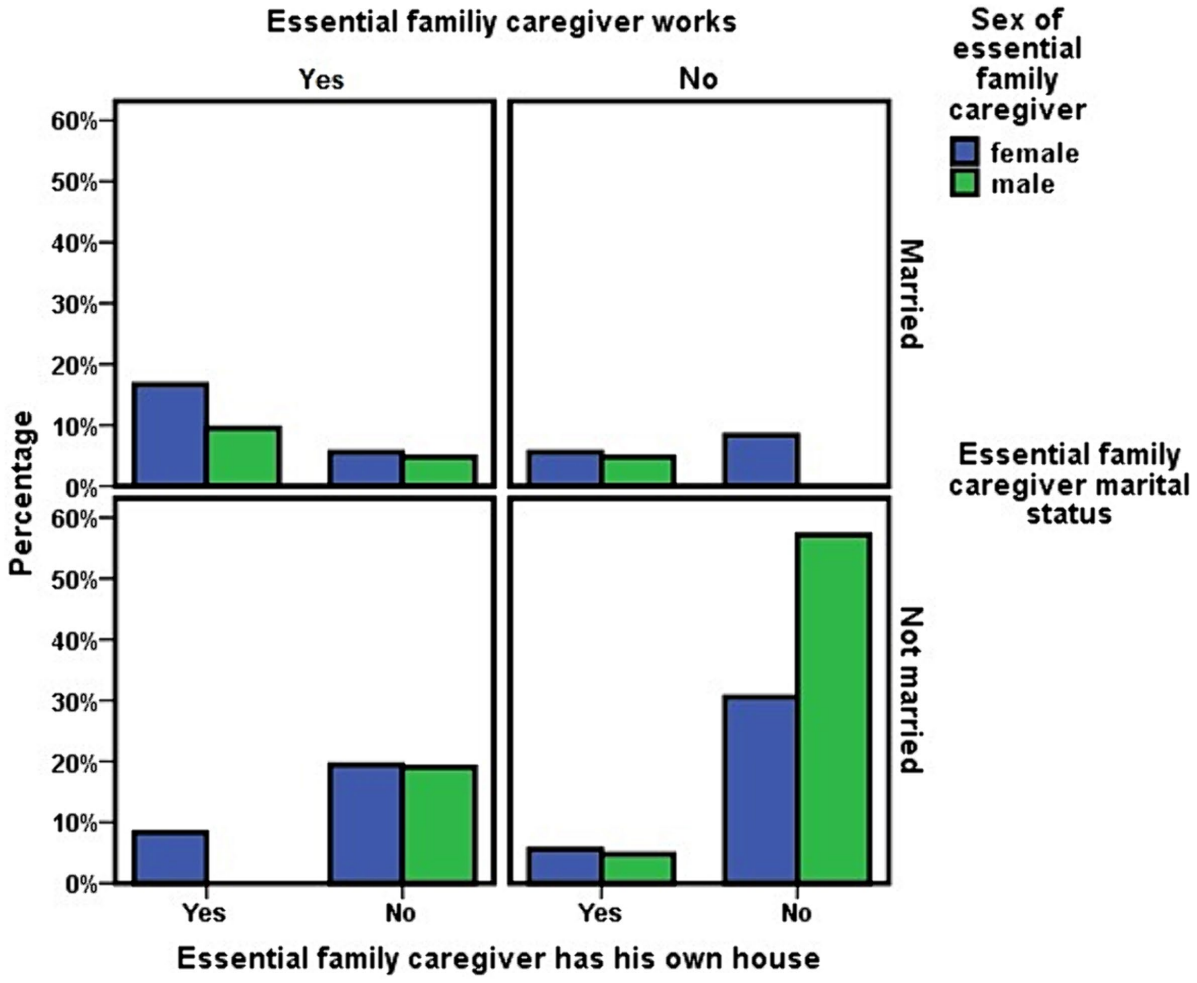
Dependency care as a factor of vulnerability and social inequality. Socioeconomic situation of the children who is the main caregiver of the dependent person.

The essential family caregiver showed a three-way interdependence between his work status, his homeownership status and his marital status. Not having his own home was associated with not working and with being unmarried. In addition, there was an association between not being married and not working (Table 6; Figure 1). Although 57.1% of the sons presented the triad of being unemployed, unmarried, and not owning his own home, compared to 30.6% among the daughters, the difference did not reach statistical significance. Neither did the differences in having a job, which was more frequent among daughters (31.6%) than among sons (12.3%), or owning his own home, a situation that 22.8% of daughters and 7% of sons had.

**Table 6:** Autonomy, dependence, and interdependence between parents with functional dependence and sons who are functionally independent caregivers.

Autonomy, dependence, and interdependence II			
Adult children: living in a dependent way despite being independently functional			
Variable	Data	Statistic value	Significance value
Sex of essential family caregiver (EFC)^a^:			
Man	21 (36.84%)	–	–
Woman	36 (63.16%)		
Essential family caregiver:			
Has a job and is married	11 (19.30%)	ꭕ^2^ = 4.275OR^b^ 3.405	*p* = 0.039CI^c^ 1.038–11.171
Has a job and is not married	14 (24.56%)		
Does not have a job and is married	6 (10.53%)		
Does not have a job and is not married	26 (45.61%)		
Essential family caregiver:			
Owns a house and is married	11 (19.30%)	ꭕ^2^ = 14.083OR = 10.389	*p* < 0.00195% CI = 2.775–38.894
Owns a house and is not married	6 (10.53%)		
Does not own a home and is married	6 (10.53%)		
Does not own a home and is not married	34 (59.64%)		
Essential family caregiver:			
Owns a home and works	11 (19.30%)	ꭕ^2^ = 4.275OR = 3.405	*p* = 0.03995% CI = 1.038–11.171
Owns a home and does not work	6 (10.53%)		
Owns a home and works	14 (24.56%)		
Does not own a home and does not work	26 (45.61%)		

Socioeconomic and demographic factors.

a Essential Family Caregiver (ECF): only child who resided in the same home as the dependent person and provided him/her with the care he/she needed in an effective, non-delegated manner, or when several children lived together, the child who assumed the principal responsibility of providing the necessary care to the dependent person.

b OR, odds ratio.

c CI, 95% confidence interval.

In this analysis of family structure, it was also observed that the number of sons (*n* = 202) that these dependent persons had was greater than the number of daughters (*n* = 193). However, the number of daughters (36) who were in the essential family caregiver role was greater than that of sons (21), with daughters being more likely to end up in the essential family caregiver role (OR = 1.794; 95% CI = 1.011–3.182). No pattern of mother cared for by daughter or father cared for by son was observed in this cohort.

The level of dependency (χ^2^ = 0.132; *p* = 0.716) or financial income (χ^2^ = 0.008; *p* = 0.930) of the ADL-dependent person did not influence whether the caregiver was a son or a daughter. And whether this essential family caregiver was male or female did not influence the ADL-dependent person’s survival at 3 years (χ^2^ = 0.030; *p* = 0.862).

However, in addition to the adult children, other people also lived with the dependent person. Of the total number of cohabitants in the same household as the dependent person (*n* = 269), 71.75% (192) were other types of cohabitants who were not adult children. Of this group of 192 persons, 165 (85.9%) were living together in a household in which the dependent person (s) and one or more children of those dependents also resided. The remainder (*n* = 27) were other persons who were not adult children, living in the same household as the dependents, with no adult children living in that household.

The mean range of cohabitants was higher when the dependents were married than when they were not married (z = −2.006; *p* = 0.045). This result was determined by male cohabitants (Kruskal–Wallis χ^2^ = 7.216; *p* = 0.007), who represented 38.2% (*n* = 63) of this group, and was not observed in relation to female cohabitants (Kruskal–Wallis χ^2^ = 0.008; *p* = 0.927), who represented the remaining 61.8% (*n* = 102). The number of residents (z = −0.776; *p* = 0.438), or whether these residents were male or female (Kruskal–Wallis χ^2^ = 0.377; *p* = 0.889), did not influence dependent survival at 3 years.

### Socioeconomic determinants and mode of coexistence of patients with functional dependence

Economic capacity is a factor that can condition the results of any study. In this study, and in relation to the functionally dependent population that makes up the Orcasitas cohort, its influence was analyzed together with the social factors that accompany these dependent persons.

By analyzing the socioeconomic and family structure data jointly, in relation to lifestyle (Tables 3, 6), we found that 54.33% (*n* = 69) of people with ADL dependence did not live with their adult children, while 45.67% did. In addition, 37% lived alone or with a partner without other cohabitants.

**Figure 2:**
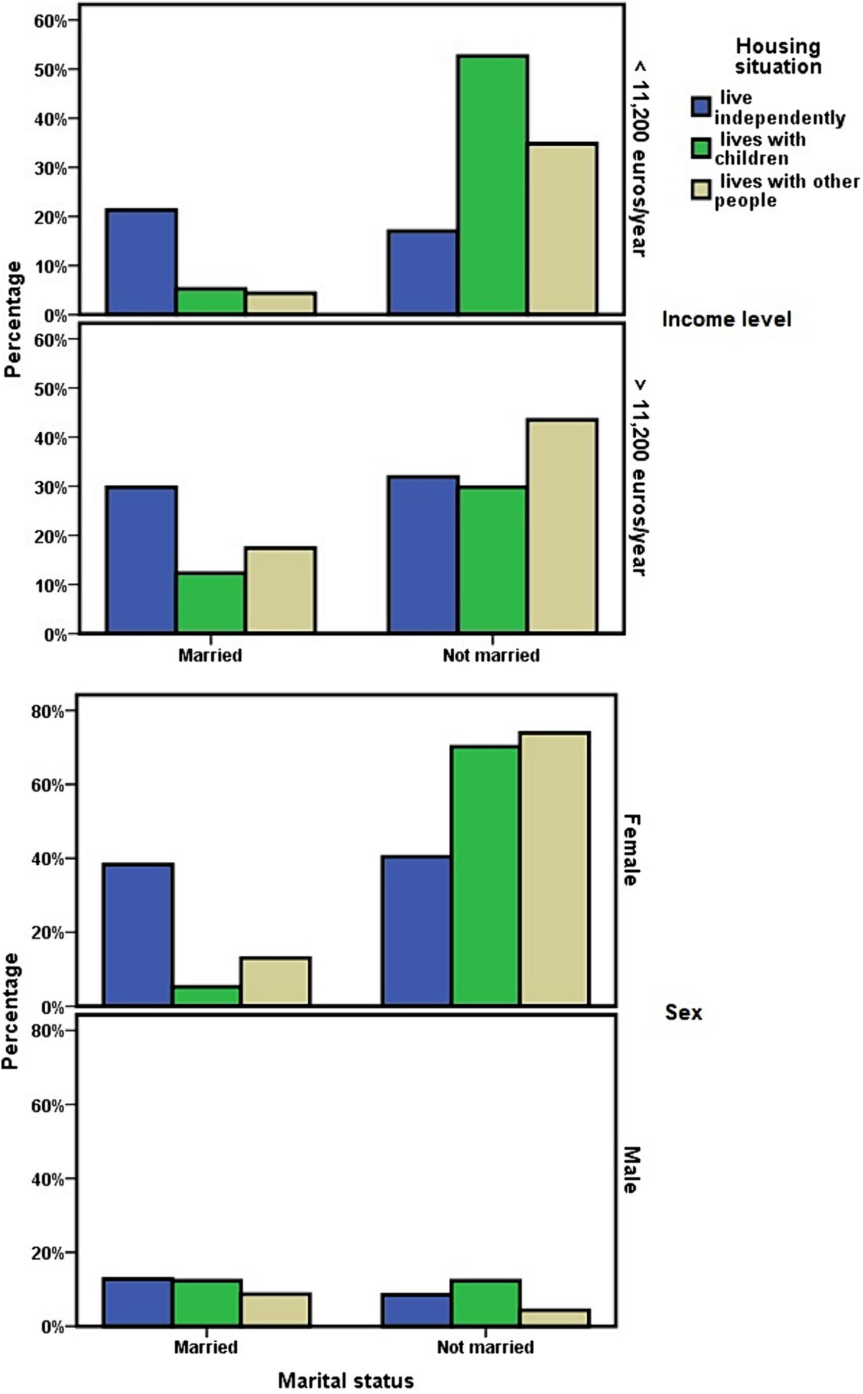
Socioeconomic determinants in relation to living independently or with children.

At the cohort level, living in a children-dependent manner was associated with the level of economic income of the dependent person (Figure 2), this dependence being more likely if they had an economic income of less than 11,200 euros/year (χ^2^ = 3.959; *p* = 0.047).

Among the ADL dependents who cohabited with their adult children, 75.9% (*n* = 44) were women, and 24.1% (*n* = 14) were men. No differences were observed depending on the married–unmarried marital status in relation to the level of dependency (χ^2^ = 0.312; *p* = 0.576) or 3-year survival (χ^2^ = 0.574; *p* = 0.449). However, it did in relation to economic income, which was lower in ADL-dependent people who did not have a partner and lived with their adult children (χ^2^ = 3.871; *p* = 0.049).

Living with adult children or other people was also associated with not being married (χ^2^ = 14.664; *p* = 0.001), a result that was maintained when the groups “living independently alone or married” were compared to “living with their adult children” (OR = 4.904; 95% CI = 2.013–11.948). When analyzing this result in relation to sex, it was observed that women with functional dependence who were married more frequently maintained the personal situation of living independently of their children. However, women who did not have a partner tended more frequently to live with their children (OR = 12.632; 95% CI = 3.312–48.178). In men, these differences were not observed depending on their marital status (Figure 2).

**Figure 3:**
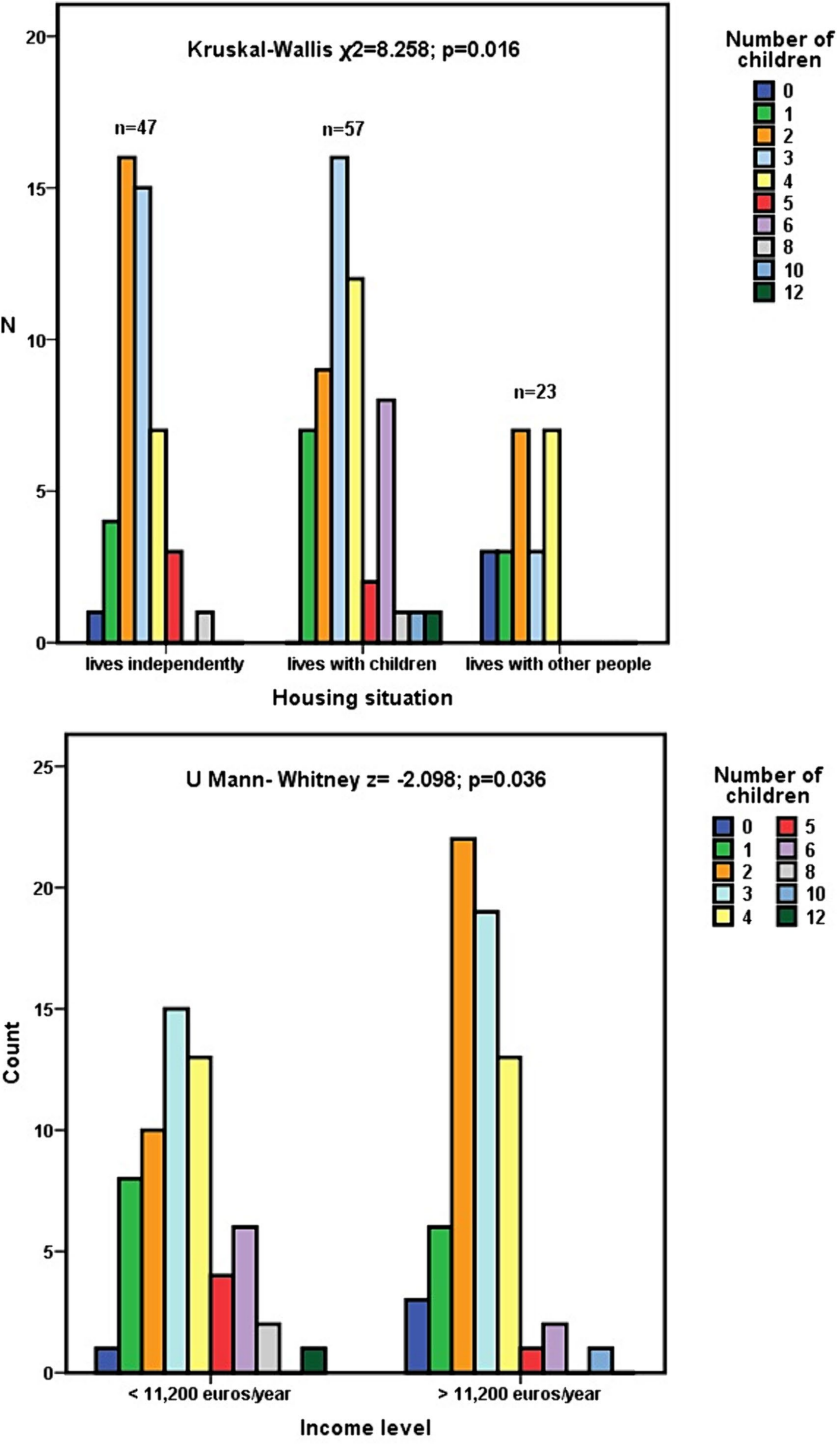
Relationship between the number of children that dependent people had and the economic level or current way of living together of those people.

In this line, having fewer children was associated with a greater probability of living with people other than children (Kruskal–Wallis χ^2^ = 8.258; *p* = 0.016; Figure 3). The number of children showed no association with the economic level of the dependent person. Dependents with an economic income of less than 11,200 euros/year had an average of 3.48 ± 2.06 children, compared to 2.78 ± 1.52 children among those with a higher income. However, among those with higher incomes, there was a significant predominance of having had two children (z = −2.098; *p* = 0.036; Figure 3).

Finally, living independently of one’s children was not associated with the score or level of dependency on the Barthel scale, and neither was it associated with the level of risk or intervention assigned within health planning.

### Age and health determinants in relation to the housing situation of dependent persons

Regarding the burden of chronicity, 23.75% (*n* = 19) of the dependent persons who lived with their children or with other persons (*n* = 80) had five or more chronic diseases. This percentage was 31.9% (*n* = 15) among people who, despite being functionally dependent, lived independently (*n* = 47). The differences between the two groups were not significant (χ^2^ = 1.007; *p* = 0.316). When these data were broken down to differentiate those who lived with their children and those who lived with other people who were not children, the differences also did not show statistical significance with respect to dependents living independently. These data reflected that 29.8% (*n* = 17) of those living with their children (*n* = 57) and 8.7% (*n* = 2) of those living with other non-children and non-partners (*n* = 23) had five or more chronic diseases (χ^2^ = 4.739; *p* = 0.094).

As to the number of drugs they had prescribed in relation to their diseases, 89.4% (*n* = 42) of those living independently (*n* = 47) and 92.4% (*n* = 73) of those living with others (*n* = 79) had five or more active principles prescribed (χ^2^ = 0.343; *p* = 0.558). Breaking down the data again for those living with people other than their partner, 89.5% (*n* = 51) of those living with their children (*n* = 57) and 100% (*n* = 22) of those living with people other than their children had five or more active principles prescribed (Likelihood Ratio 4.439; *p* = 0.109).

When the relationship between polypharmacy (consumption of five or more drugs), the number of chronic diseases, and age was analyzed, it was observed that 83.3% (*n* = 10) of the ADL-dependent persons who were less than 80 years old (*n* = 12) and 92% (*n* = 104) of the ADL-dependent persons who were 80 years old or older (*n* = 113) were included in the Polymedicated Patient Protocol (χ^2^ = 1.024; *p* = 0.312). When the cutoff point was set at 90 years, 90.5% (*n* = 76) of ADL-dependent persons who were less than 90 years old (*n* = 84) and 92.95% (*n* = 39) of persons who were 90 or older (*n* = 42) had five or more drugs prescribed (χ^2^ = 0.199; *p* = 0.655).

The burden of chronicity in dependent persons who were less than 80 years old showed that 16.7% (*n* = 2) of those who were less than 80 years old (*n* = 12) and 28.1% (*n* = 32) of those who were 80 years old or older had five or more chronic diseases (Fisher’s exact statistic *p* = 0.511). These percentages were 24.7% (*n* = 21) among those under 90 years of age (*n* = 85), and 31% (*n* = 13) among those 90 or older (*n* = 42, χ^2^ = 0.559; *p* = 0.454).

### Relationships between the level of functional dependence, income level, and the availability of assistants for housework and personal care

With respect to the assignment from Social Services, or through private hiring, of assistants for household tasks or personal care, in the Orcasitas cohort, no association was observed between the availability of such services and a higher level of dependency. Of the patients in this cohort, 27.6% did not have any type of assistant for carrying out household tasks or maintaining basic personal care, nor did they have an internal caregiver. This situation was also observed in 26% of the people with severe dependency, especially in the group of married people living independently, where half of them had no such aids.

**Figure 4:**
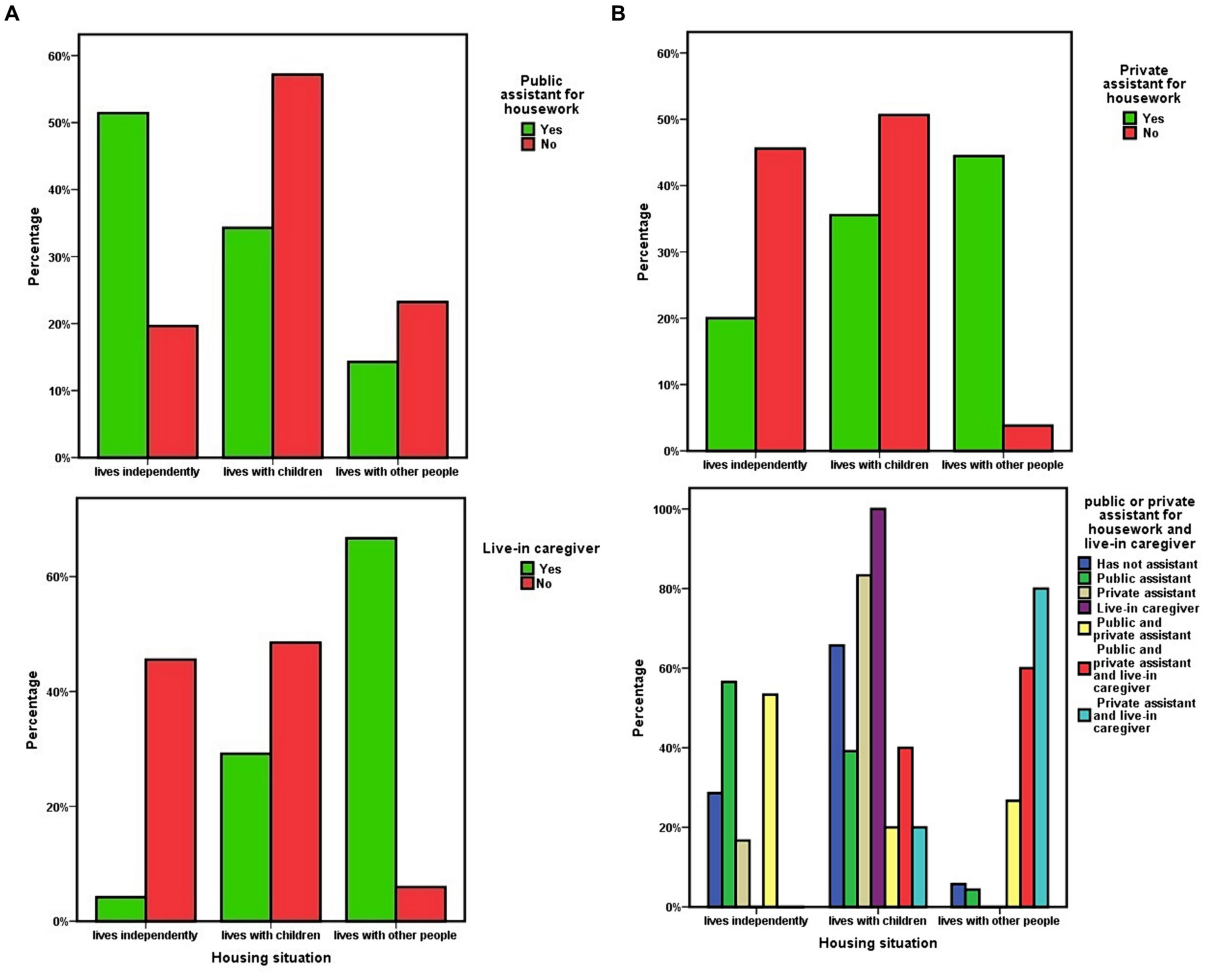
**(A)** Housing situation of dependent people and availability of public assistant for housework or live-in caregiver. **(B)** Housing situation of dependent people and availability of a private assistant for domestic tasks. Housing situation and coexistence of multiple non-family caregivers.

Disaggregating the data from this study in relation to the mode of cohabitation, it was observed that, despite not having a greater functional dependency, the subgroup of dependent population that “lives with others” more frequently had an live in caregiver (Kruskal–Wallis χ^2^ = 50.673; *p* < 0.001) or a private assistant for domestic tasks (Kruskal–Wallis χ^2^ = 31.886; *p* < 0.001). It also had more than one type of assistant, Table 5 and Figure 4 having less frequently a public assistant (Kruskal–Wallis χ^2^ = 13.336; *p* = 0.001). In this subgroup, the live-in caregiver was the person with whom 69.7% (*n* = 16) of its members lived exclusively.

The availability of assistants for housework or of an live-in caregiver was not associated with the level of economic income in the Orcasitas cohort. Regardless of the mode of cohabitation, having an assistant or caregiver was not associated with greater survival at 3 years, a result that was maintained when only the severe dependency population was considered.

### Relationship between loss of functional independence due to mobility limitations, level of dependence, and survival at 3 years

The presence of functional dependence due to limitations that restrict mobility was assessed by recording the use of a wheelchair, walker, and/or crutches/cane.

The use of crutches/cane was more frequent among persons with moderate dependence than among persons with severe dependence (χ^2^ = 12.885; *p* < 0.001). Crutches/cane were used by 52.8% (*n* = 47) of persons with moderate dependence and 18.4% (*n* = 7) of persons with severe dependence. Wheelchair use was more frequent among persons with severe dependence (47.4%; *n* = 18) than among persons with moderate dependence (25.8%; *n* = 23), χ^2^ = 5.644; *p* = 0.018. However, the use of a walker was not significantly associated with the level on the Barthel scale.

At cohort level, the use of crutches or a walker was not associated with survival, but the use of a wheelchair was associated with a lower probability of survival in this 3-year period (OR = 0.398; 95% CI = 0.186–0.853). Among users of this mobility device, 56.1% (*n* = 23) died in this period.

**Figure 5:**
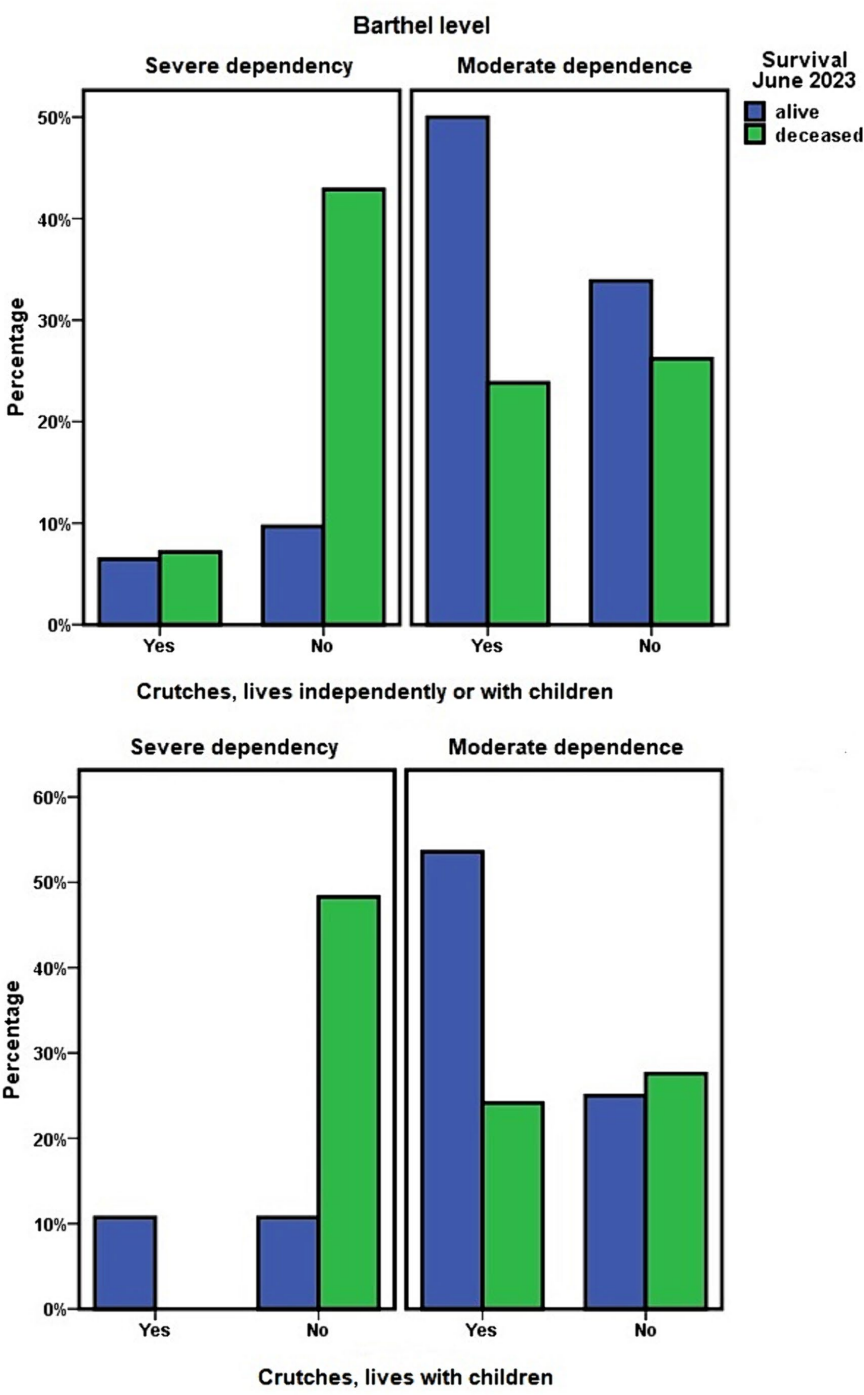
Housing situation of dependent people, use of crutches, and 3-year survival.

However, in relation to crutches/cane, when the groups “living independently” and “living with adult children” were analyzed together, their use was associated with survival at 3 years (OR = 2.892; 95% CI = 1.268–6.596). However, it did so in a direction not expected: 72.9% of dependent persons using crutches/cane were still alive at 3 years of follow-up, compared to 48.2% among ADL-dependent persons who did not use them at the start of this study (Figure 5).

Finally, within the “living with children” group, the use of crutches/cane was associated with less functional dependence (OR = 0.120; 95% CI = 0.030–0.484) and greater survival at 3 years (OR = 5.657; 95% CI = 1.792–17.854). In this 3-year period, 68.75% (22) of the dependent persons who did not use crutches/cane died, compared to 20% (*n* = 7) of those who did use this type of mobility aid (Figure 5).

The use of a walker was not associated with survival in any of the combinations analyzed.

In *post-hoc* analyses, the level of dependence explained 10.9% of the variance in survival (R^2^ = 0.109, adjusted R^2^ = 0.102, t = −3.910; *p* < 0.001). Wheelchair use explained 4.5% of the variance in survival in this cohort (R^2^ = 0.045, adjusted R^2^ = 0.038, t = −2.435; *p* = 0.016).

### Housing situation, economic capacity, number of children, and survival after 3 years

Comparing the different groups of ADL-dependent persons according to their housing situation, an analysis was made of the possible differences associated with the housing situation of living independently, living with their children, or living with other persons. Among the ADL-dependent persons living independently, either alone or with a partner, 28% (*n* = 13) died during the 3 years of follow-up. Among the ADL-dependent persons who lived with their children or with other persons who were not their children, this percentage was 48.75% (*n* = 39). Regarding the level of functional dependency, no significant differences were observed between the two groups (χ^2^ = 1.511; *p* = 0.219 and OR = 0.600; 95% CI = 0.264–1.360).

**Figure 6:**
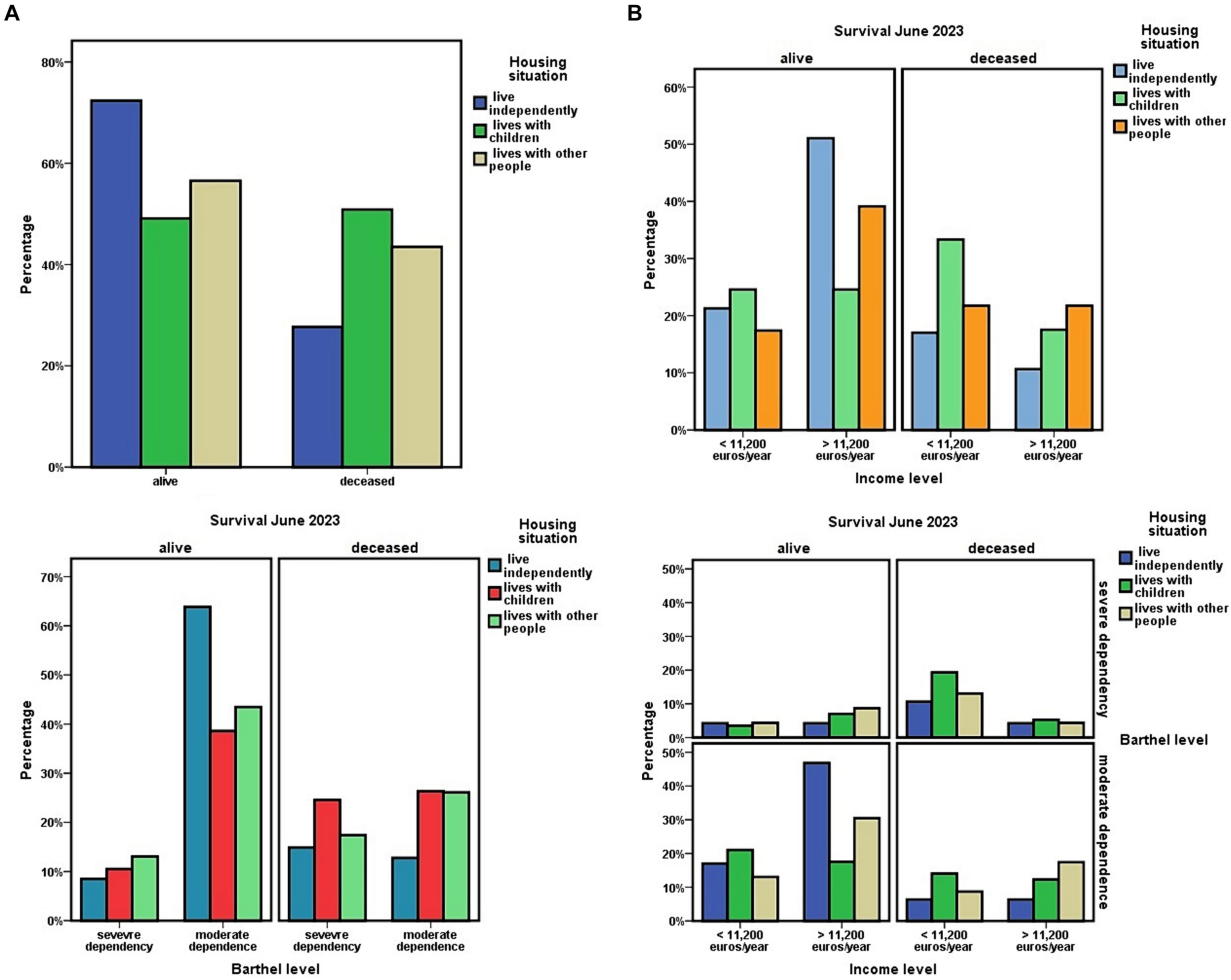
**(A)** Three-years survival in relation to housing situation of dependent people, and level of dependency. **(B)** Three-years survival in relation to housing situation of dependent people, and level of economic income. Relationships between housing situation of dependent people, level of dependency, level of economic income, and three-years survival.

As for differences between living independently or living with their adult children, 51% (*n* = 29) of ADL-dependent persons who lived with their adult children died in the period 2020–2023, compared with 28% (*n* = 13) among persons who lived independently (χ^2^ = 5.768; *p* = 0.016). A higher mortality risk was observed among functionally dependent persons living with their children, compared with ADL-dependent persons who did not live with their children and lived alone or with their partner, OR = 2.709; 95% CI = 1.189–6.172 (Table 5; Figure 6).

In a longitudinal study, the time variable modifies many aspects that should be controlled. Given the age of this population group and their ADL dependence, a percentage of dropouts was to be expected, due to personal circumstances that obliged the dependent person to live with children residing in other localities or due to admission to a nursing home. For this reason, in the survival analysis using Cox regression with respect to the housing situation variable, only those ADL-dependent persons who completed the study period in their home, or who died while that home was their habitual residence, were included.

Previously, a contrast was performed to assess the association between the variables included in the study and survival. This analysis showed that sex (t = 2.675; *p* = 0.008; 95% CI = 0.073–0.487), level of economic income (t = −2.745; *p* = 0.007; 95% CI = −0.404 to −0.065), level of functional dependence (t = −3.910; *p* < 0.001; 95% CI = −0.534 to −0.175), and living independently (t = 2.367; *p* = 0.019; 95% CI = 0.035–0.387) were associated with survival at 3-year follow-up. The factors being male, having a financial income of less than 11,200 euros/year, having severe functional dependence, and living with children or others were associated with lower survival at 3 years.

Age, being over 80 years old or 90 years old, being married, or being widowed, were not associated in this study with survival. There were also no significant differences in survival at 3 years for living with children as a couple or without a partner (χ^2^ = 0.112; *p* = 0.738). Educational level did show an association with survival (t = 2.151; *p* = 0.033; 95% CI = 0.023–0.554), but it was not used in the Cox regression or in the *post-hoc* analyses because the percentage of people with education was very low.

The number of children had by the ADL-dependent persons in this cohort was not associated with greater or lesser functional dependence on their parents, nor with survival at 3 years. The number of cohabiting children, or whether the cohabitation was with children older or younger than 55 years of age, the pre-retirement age in Spain, was not associated with survival. Nor was it associated with the dependent’s caregiver child not owning his own home and living in the dependent’s home or being married or single. These results were not modified when these parameters were adjusted for the level of income of the dependent person.

In the bivariate analysis by linear regression, the level of economic income was associated with survival at 3 years (HR = 0.40; Exp(B) = 0.596; 95% CI = 0.459–0.774). Among dependents with incomes below 11,200 euros/year, 53.3% (*n* = 32) had died at 3 years of follow-up, compared to 29.9% (*n* = 20) among those with incomes above 11,200 euros/year (Figure 6B).

When adjusted this parameter according to the situation of living independently or not, presenting both situations, having better economic capacity and living independently, was associated with greater survival at 3 years (HR = 0.52; Exp(B) = 0.471; 95% CI = 0.234–0.935).

**Figure 7:**
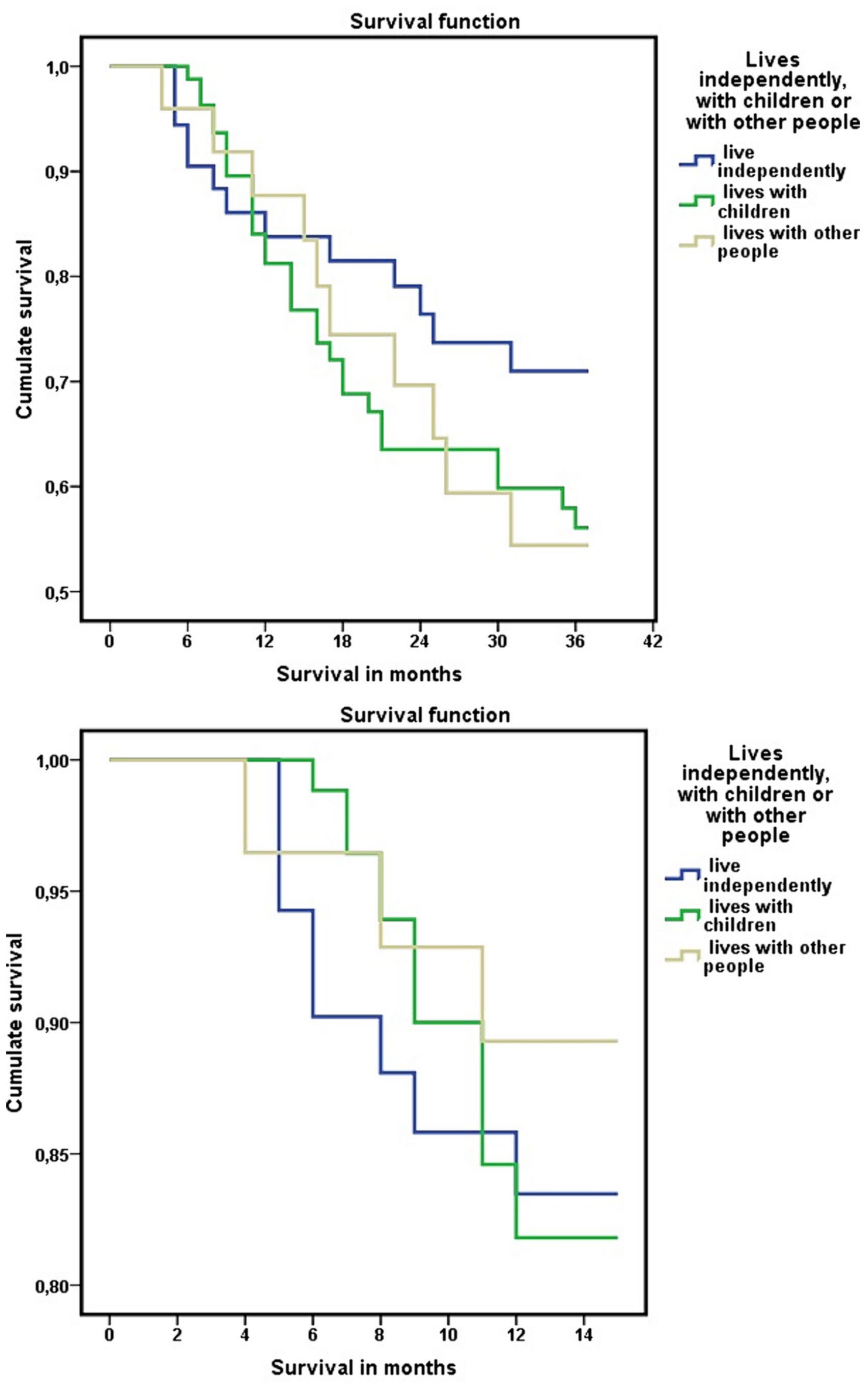
Differences in mortality at 3 years in the functionally dependent population of the Orcasitas cohort according to living independently, with their children or with other people, adjusted for sex, Barthel index level, and economic capacity (Cox regression).

Using Cox regression, when adjusting the data for housing situation according to sex, level of income, and level of dependency according to the Barthel index, a higher mortality at 3 years was observed among those who lived with their adult children or with other people (HR = 1.345; 95% CI = 1.010–1.792; Figure 7).

Events, such as the COVID-19 pandemic, occurred in the period included in this longitudinal study that justified a differential analysis of the results. Although overall 3-year mortality was higher among those living with their children or others, this result was not clear during the first year of the COVID-19 pandemic.

Analyzing separately the data between June 2020 and June 2021, after confinement for the COVID-19 pandemic, mortality was higher among those who did not live with their children, although without reaching statistical significance. These minimal differences between the three groups disappeared 12 months after the end of home confinement for the COVID-19 pandemic. From that date, June 2021, this trend was reversed, and mortality began to be higher among those who were not living independently, a situation that continued from that date until June 2023.

In the *post-hoc* analyses, using stepwise linear regression, these associations were maintained. Living independently and living independently having better economic capacity were associated with survival at 3 years (Table 7). This result was also observed by Wald’s chi-square test (χ^2^ = 4.276; *p* = 0.039) and in multiple comparisons analysis with Bonferroni correction (*p* = 0.031; 95% CI = 0.02–0.47).

**Table 7:** Survival at 3 years of follow-up.

*Post-hoc* regression analysis. Orcasitas cohort									
Model summary									
Model	R	*R* ^2^	Adjusted *R*^2^	Std. error of the estimate	Change statistics				
					Change in *R*^2^	Change in F	gl1	gl2	Sig. change in *F*
1	0.207^a^	0.043	0.035	0.485	0.043	5.601	1	125	0.019
2	0.296^b^	0.088	0.073	0.475	0.045	6.108	1	124	0.015

ANOVA^c^					
Model	Sum of squares	gl	Mean square	F	Sig.
1					
Regression	1.317	1	1.317	5.601	0.019^a^
Residual	29.392	125	0.235		
Total	30.709	126			
2					
Regression	2.697	2	1.348	5.969	0.003^b^
Residual	28.012	124	0.226		
Total	30.709	126			

Coefficients^c^							
Model	Unstandardized coefficients		Standardized coefficients	*t*	Sig.	95% Confidence interval for B	
	B	Std. Error	Beta			Lower bound	Upper bound
1							
(Constant):	1.066	0.151		7.035	0.000	0.766	1.366
Housing situation	0.211	0.089	0.207	2.367	0.019	0.035	0.387
2							
(Constant)	1.436	0.211		6.805	0.000	1.019	1.854
Housing situation	0.181	0.088	0.178	2.052	0.042	0.006	0.356
Income level	−0.211	0.085	−0.214	−2.471	0.015	−0.380	−0.042

*Post-hoc tests performed using linear regression analysis in relation to the housing situation of living independently of children or living with children and economic level. Stepwise regression analysis.*

a Predictors (Constant): housing situation (live independently or lives with children).

b Predictors (Constant): housing situation, income level.

c Dependent variable: survival in June 2023.

On the other hand, within the *post-hoc* analyses and in the possible development of models, it was observed that the housing situation explained 4.3% of the variance of survival at 3 years in the functional ADL-dependent population of the Orcasitas cohort. This percentage increased to 8.8% when income level was included in the model.

### Age and marital status as factors in the analysis of housing status and survival

In the follow-up of this cohort over a 3-year period, being 80 years of age or older (*n* = 114) was not associated with a lower probability of survival. During this period, 41.2% (*n* = 47) of the dependent patients over 80 years of age died, compared to 33.3% (*n* = 4) among those under 80 years of age (OR = 1.403; 95% CI = 0.399–4.930). Among those over 90 years of age (*n* = 42), 52.1% (*n* = 22) died, compared to 35.1% (*n* = 30) among those under 90 years of age (OR = 2.017; 95% CI = 0.951–4.275).

In *post-hoc* analyses, using linear regression, age explained 9.6% of the variance in housing status (*R*^2^ = 0.096; adjusted *R*^2^ = 0.089; t = 3.654; *p* < 0.001). However, performing these analyses in relation to being 80 or older or 90 or older, no significance was observed.

The housing situation in relation to age showed that among ADL-dependent persons under 80 years of age, 58.3% (*n* = 7) lived independently, while among persons over 80 years of age, this percentage was 35.1% (*n* = 40). Among the dependent persons living with their children or with persons other than their children or partner, 41.7% (*n* = 5) were under 80 years of age and 64.9% (*n* = 74) were 80 years of age or older (OR = 2.590; 95% CI = 0.772–8.689). When age 90 years was used as the cutoff point for this analysis, 78.6% (*n* = 33) of those aged 90 years or older and 55.3% (*n* = 47) of those aged less than 90 years lived with their children or others. The remaining 21.4% (*n* = 9) and 44.7% (*n* = 38), respectively, lived independently (OR = 2.965; 95% CI = 1.264–6.950).

As for marital status, 9.5% (*n* = 4) of the persons older than 90 years and 41.2% (*n* = 35) of the dependent persons younger than 90 years had a partner (OR = 6.650; 95% CI = 2.176–20.323). Being widowed was the marital status of 83.3% (*n* = 35) of the dependent persons older than 90 years and 55.3% (*n* = 47) of those younger than 90 years (OR = 0.247; 95% CI = 0.099–0.619).

When combining marital status and housing situation, 73.2% (*n* = 60) of the ADL-dependent persons who are widowed lived with their children or with other persons, while 55.6% (*n* = 25) of the married persons lived independently (OR = 0.293; 95% CI = 0.137–0.630).

In the analysis of survival in relation to housing situation, 72.3% (*n* = 34) of functionally dependent persons who were living independently (*n* = 47) and 51.2% (*n* = 41) of those living with their children or others (*n* = 80) were alive at the end of the 3-year follow-up period (OR = 2.488; 95% CI = 1.146–5.400).

In the *post-hoc* analyses, survival at 3 years of follow-up showed no association with being older or younger than 80 years of age. Neither was there any association with being older or younger than 90 years of age. These results were in line with those observed in the conventional analysis. In this analysis, 41.2% (*n* = 47) of those aged 80 years or older (OR = 1.403; 95% CI = 0.399–4.930) and 52.4% (*n* = 22) of those aged 90 years or older (OR = 2.017; 95% CI = 0.951–4.275) had died in this 3-year period, compared to 33.3% (*n* = 4) and 35.3% (*n* = 30) in those aged under 80 and 90 years, respectively.

### Impact of COVID-19 pandemic confinement on functional dependence, according to the dependent person’s living situation, and survival at 3 years

Confinement to the usual home for several months during the COVID-19 pandemic showed that the degree of response to this stressor in relation to functional capacity was variable among dependent persons. One group of these persons (43.3%, *n* = 55) improved their functional capacity during confinement for the COVID-19 pandemic and were no longer dependent. This result was analyzed by dependent population groups, differentiating, according to their housing situation, those living independently, with children, or with other people.

Among those living independently, either alone or with a partner, 59.6% (*n* = 28) not only improved their functional capacity but did so with such intensity that they ceased to be dependent. That is, after confinement, these persons had a score above 60 on the Barthel index.

When the impact of confinement was analyzed among dependent persons who lived with other persons, besides their partner, this functional improvement was of lesser intensity, being present in 33.8% (*n* = 27). This level was similar among those who lived with their children (35.1%, *n* = 20) and those who lived with people other than their children (30.4%, *n* = 7).

Analyzing these data jointly with those living independently, living independently was associated with a higher level of functional improvement during confinement (χ^2^ = 8.186; *p* = 0.017). When functionally dependent persons lived independently, the likelihood that during confinement to the usual home during the COVID-19 pandemic, their functional capacity improved was greater than that observed when living with adult children or others (OR = 0.346; 95% CI = 0.164–0.728).

The improvement in functional capacity also influenced survival. Lower survival at 3 years was observed among dependent persons who lived with their children and who, during confinement in the usual home, did not improve their functional capacity or who did so without achieving a cessation of dependency (χ^2^ = 6.682; *p* = 0.035).

In the latter analysis, among those who improved their functional dependence to the point of no longer being dependent, no differences were observed with respect to survival at 3 years for whether they were living independently or living with their children or others (OR = 1.544, 95% CI = 0.454–5.248). Among those who ceased to be dependent after confinement, 78.6% (*n* = 22) of those living independently and 70.4% (*n* = 19) of those living with others were still alive at 3 years.

However, among those who did not have the same response to home confinement and either did not improve their functional capacities or did not improve to such an extent that they were no longer considered a dependent population, differences in survival were observed. In this group of people who remained functionally dependent (Barthel ≤60) after home confinement for the COVID-19 pandemic, 63.2% (*n* = 12) of those living independently and 62.5% (*n* = 10) of those living with other people who were not adult children survived during the follow-up period of this study. However, among those living with their children, this percentage was 32.4% (*n* = 12).

## Discussion

The first wave of the COVID-19 pandemic showed a high mortality rate among older people who were institutionalized in nursing homes (17). This situation made us question what was taking place with our older people in their homes, particularly with older people with functional dependence. Aspects such as how they were being cared for, how their lives had changed, how the confinement had affected their personal situation, and what this might imply for their survival became relevant issues that needed to be answered.

Although Spanish legislation includes the obligation of care from children to their parents, the social reality sometimes makes it difficult to make these functions compatible or to develop them. This situation leaves the dependent person in a social limbo, which needs to be addressed (18). And this situation worsened during the nationwide COVID-19 lockdown.

Before 1970, Orcasitas was a neighborhood of shantytowns or substandard housing in the city of Madrid. To solve the poor living conditions of these people, between 1970 and 1990, these people were rehoused in newly built quality housing. As a neighborhood, it has been classified as marginal, poor, working class, or suburb.

Based on this overall environment, the results of this study suggest that feedback from the socioeconomically disadvantaged environment model is occurring, which favors the perpetuation of the model and the creation of a ghetto (19–21).

It was expected that the adult children would have better social conditions than the parents. On a base of older parents, mostly women, with little or no cultural level and low economic income, who obtained social housing when the state oversaw this function, the result was probably not what was expected.

Some of these children were unable to emancipate themselves for some reason and continued to live with their parents. Another part had to return to the parental home when their life project as independent persons failed (unemployment, divorce, separation), carrying their descendants with them in this return to the parental home. And another part of these adult children was married; they had their children, but they reached an agreement with their parents to remain in their parents’ home.

All these people were supporting the perpetuation of the model, the failure of a generation in its attempt to progress, and the maintenance of the social status in which their parents were situated (22). With a worsening trend that should be considered in political and social planning.

In this environment, the housing situation of people with ADL dependency who resided in the community was heterogeneous. There were many patterns of cohabitation, and many people with different or no kinship participated in that cohabitation. Some dependent households had high rates of residents’ occupancy, while in others the number of adult children who kept their parents’ home as their home revealed the existence of complex interrelationships beyond dependency.

The presence of common characteristics among the people who participated in them suggested the existence of a pattern (23). Furthermore, by occurring in a specific context, functional ADL dependence on their parents, and in a specific environment, a marginal neighborhood in the city of Madrid, the repetition of this pattern constituted a network.

This network activated heuristics that impeded the essential caregiver’s own personal development (21–24).

On the one hand, from the family perspective, prone in the Mediterranean area to caring for older parents, obligations and a sense of reciprocity were created, caring for having been cared for.

On the other hand, by imitation, since it was a practice that was shared by other neighbors, this situation was normalized and taken for granted (21, 25). Finally, due to the needs of the caregivers themselves and their family members, since they do not have their own home, job, and/or partner, it ended up being the solution to their problems.

This combination of situations generated intergenerational dependency and created poverty traps (23). Traps from which it is very difficult to escape, given the high age of the essential caregivers and their current and previous social situation.

And these results allow us to predict that the social and economic future of many of the people who play the role of essential caregivers of the ADL-dependent persons in this study will be worse than that of their parents. With a high risk of ending up under the poverty line (21, 22).

However, observing the reality from the perspective of ADL-dependent persons, it could also be intuited that there was a network that explained the results in function of various factors. Factors that formed patterns in this final stage of life. This network, this pattern, would justify why this evolutionary course was independent of the burden of chronic diseases and the number of drugs prescribed for their diseases. And that it was also independent of the level of functional dependence.

ADL-dependent people, like all people, respond to situations by making changes, and probably the aim of that heuristic was to maintain independence (26). And, when this was no longer possible, to ensure being cared for by others.

This network was made up of multiple threads, and, despite its complexity, the pieces ended up fitting together within a framework that is likely to be common in similar populations. The common threads in this network were income level, marital status, and age.

Having a higher level of income made it possible to live independently, but this was modulated by marital status and by an unavoidable factor, life expectancy.

Life expectancy is higher in women than in men (27). During the 3-year follow-up period, proportionally more men died than women. In this cohort, being widowed and having an older age implied a higher probability of ending up living with children or with other people. Above the age of 90, having a partner was an exception.

And being a widow became a risk factor for living with other people. This result may have been influenced by the fact that the economic income of widowed women was lower than that of married women. The economic factor could justify, among other factors, why this result was not observed among men.

So, an evolutionary course appeared to be established, in which living independently was more frequent than the younger one was, in relative terms, since the mean age in this cohort was 86 years. And, when one had a partner and a higher level of economic income.

And it was not the level of functional dependence that determined whether ADL-dependent persons had to live with their children or with other people, but the loss of a partner and age.

However, the residential solution adopted by this very older dependent population may have been influenced by other factors, including the absence of family members with whom to live (28). This factor has been reported in various studies (29), in which, as observed in this study, a smaller number of children increases the probability of living with other people, generally an internal caregiver hired for this purpose.

On the other hand, there is a tendency to think that when people get older, they end up moving in with their children. The results obtained through this study reflect that widowed dependent women who live with their children do not generally do so at their children’s home, but rather it is the children who live at their mother’s home.

When this was not the situation, it cannot be excluded that in a certain percentage of cases, leaving with the children was a personal decision (30), not associated with dependency or comorbidities, a decision in which loneliness may have played a role.

Other factors were also essential threads in this network. The number of children these people had (31), having a daughter, the fact that the first child they had was a daughter (32), and, unfortunately, the successive socioeconomic and personal crises that affected their children ended up giving form to a model of care for ADL-dependent persons.

With respect to children, although the stereotype that people with low socioeconomic resources have a high number of children may be maintained, in this cohort the differences in the number of children in relation to economic level were not significant. This result may be justified by the fact that the economic income of this population group is low overall in relation to the mean for the city of Madrid. However, it was more frequent for dependent persons with higher incomes to have two children and for persons with lower incomes to have three children (33).

The loss of a partner implies a loneliness that increases fragility and vulnerability and motivates changes (27). Age conditions a global deterioration beyond the level of dependence, which also motivates to make changes. Older age and living with other people are factors that have been associated with less personal autonomy (34). Age and loss of a partner were risk factors for ending up cohabiting with other people in this study. However, other factors are also involved.

The findings in this cohort showed that most of the ADL-dependent persons lived with people other than their partners, and that this cohabitation was probably a necessity, which did not reflect the level of dependency that they had recognized. However, it did reflect other data, such as the fact that most of the live-in caregivers were employed by this population subgroup. Or that they had a greater need to use a wheelchair when moving around.

Based on these characteristics, it was possible to establish a specific group of ADL dependents, made up of those who were cared for exclusively by an internal caregiver. This situation was present in one out of every 10 ADL dependents in this cohort. And it did not respond to economic criteria, since this subgroup occupied the second place with the lowest economic income, behind that of single ADL dependents living with their children. In other words, the ADL-dependent persons who had a live-in caregiver were not those with the greatest purchasing power.

The presence of internal caregivers providing care to people with low economic resources indicated that this care was a real need that the level of dependency did not reflect. It could be observed that the percentage of persons with severe dependency within this subgroup was like that observed in the subgroup of dependent persons living alone. The existence of this need was reinforced by the parallel situation of a greater presence of private caregivers in this subgroup. Then there was another factor, undetected by the level of dependency, which meant that two apparently similar groups in terms of dependency had different care needs. This result has already been observed in other studies (35). This subgroup of dependent people who lived with people other than their children also had less personal autonomy, reflected in a greater use of a wheelchair. And these care needs were not visualized by health indicators. Care needs that reflected invisible situations that transcended beyond functional dependence and led to higher mortality among these people.

This model of care for the dependent person, together with that represented by the Essential Family Caregivers, provides round-the-clock care for dependent persons, saving costs to the social and healthcare system.

Being cared for implies that costs are generated to meet this need. When the social system does not assume these costs, it is the dependent persons themselves and their families who must take care of these costs. In socioeconomically disadvantaged environments, such as Orcasitas, and considering that almost half of the population of this cohort has a level of income that the health and social system equates to poverty, assuming this cost is a challenge. There is a need for a great deal of justification to dedicate a portion of scarce resources to meet this need.

And this circumstance probably partly justifies the unequal distribution of assistants for housework and personal care observed in this study. Because Social Services only partially funds the cost of housework assistants. The distribution of these assistants did not respond to criteria of functional dependence. This result could be implied by not being able to assume the cost of this assistance, a situation that can sustain social inequalities and inequities in comparison with the care given to patients with similar characteristics who are institutionalized (29).

These inequalities were evidenced by the fact that one in four people with severe functional dependency had no support resources from Social Services. And half of the people in this situation were married dependents who lived with their partner. This situation meant that the partner was the main caregiver for one out of every five married dependent persons, a partner who was also older adult and sometimes also dependent.

This study also made it possible to detect the existence of people who lived alone despite being severe dependency, people who, in some cases, had no such resources. Finally, another part of the people with severe dependency who were not assigned this assistance by Social Services lived with their adult children, making the adult children the full-time caregivers of these people and affecting their own social situation (36).

Although various studies associate having social resources for housework with the educational and economic level of the ADL-dependent person (12), in this study this association was not observed. It is likely that the low educational and economic level of this cohort influenced this result.

On the other hand, in this cohort, the presence of private assistants and internal caregivers hired to perform housework and provide care implied a real need for help. The non-association of these resources with the level of functional dependence could be evidence of the existence of other limitations in these individuals, justifying this need. This existence is supported using mobility aids, which in this study showed differences according to the housing situation.

Although psychological factors were not analyzed in this study, it would also be necessary to address in other studies to what extent the manner of confronting dependence transforms itself into a factor of dependence, justifying the differences observed.

The limitations associated with functional dependence often require assistance by third persons in the performance of basic activities of daily living. This assistance is often complemented using mobility assistance devices. Maintaining mobility is a relevant factor in quality of life and survival.

In relation to functional dependence, there is a complexity in analyzing the relationship between survival and the use of mobility assistance devices. This complexity is because this use can be simultaneous in the same patient, depending on the mobility situation faced. Or sequential over time, as dependence worsens, progressing from the use of crutches/cane to the use of a walker, and from this to the use of a wheelchair. The data obtained in this study suggest a sequential use over time, with the use of crutches/cane predominating when dependence is moderate, and the use of a wheelchair when dependence is severe. The use of a walker would be an intermediate step used by people with both moderate and severe dependence.

Instability and immobility have been linked as causes of needing a wheelchair. Being in a wheelchair increases the restriction of living space and represents a preliminary stage to bedridden (37). Loss of mobility resulting from wheelchair dependence leads to progressive homebound. And being homebound is associated with an increased risk of death regardless of functional impairment and comorbidities (38).

In this study, the use of a wheelchair was associated with increased mortality (37), and the use of crutches/cane was associated with increased survival. In relation to crutches/cane, this result was probably influenced by the fact that its users were generally patients with moderate dependence, who showed greater survival. However, greater survival was also observed among people with moderate functional dependence who used crutches/cane and who lived with their children. And in this group, the observation that survival was higher among those who used crutches/cane than among those who did not use this aid does not allow excluding other causes. Causes that will have to be analyzed by means of specific studies.

Although several studies highlight the role of rehabilitation in healthy aging (39), currently the non-institutionalized population that has severe functional dependence does not have access to these services at home. And this situation is especially relevant among wheelchair users, whose use has been associated in this study with higher mortality. These results advise that health planning focused on healthy aging includes the development of specific physiotherapy and occupational therapy services for the functionally dependent population, which improve their quality of life.

In relation to survival and analyzing the 3-year follow-up period, living with adult children apparently had a dual pattern. During the first year of the COVID-19 pandemic, living with adult children was to a certain extent a protective factor. This was true both during the period of home confinement during the first wave of COVID-19 and during the successive waves of COVID-19 after home confinement. Although the cause of death was not recorded, several factors could have influenced this result. Among them is the loss of follow-up of chronic pathologies by the health system due to having to dedicate available resources to the care of successive waves of the COVID-19 pandemic. This situation may have contributed to the deterioration of an already fragile and vulnerable population (40, 41). And perhaps also because dependent persons who did not have other family support and did not have or lost social resources (day centers, caregivers) were more exposed to these waves of COVID-19 and supported a higher level of stress, which may have contributed to this outcome (28).

When high vaccination coverage against COVID-19 was achieved and healthcare was normalized, living with one’s children was no longer a protective factor. And a reality was beginning to emerge in which the level of dependency, modulated by the economic level, were relevant factors for survival. And where maintaining personal autonomy, reflected in the ability to live independently of one’s children or other people, played a relevant role in these results.

After confinement to the usual home due to the COVID-19 pandemic, it was observed that almost half of the persons in this cohort had less functional dependence, to the point of no longer being dependent (Barthel >60). However, among dependent persons living with their children, failure to achieve this level of functional improvement was associated with lower survival.

Within the analysis of factors associated with survival, the level of economic income has been associated with the maintenance of personal autonomy and independence in the older persons (42). And, with an increase in life expectancy (43, 44). These results were also observed in this study. Greater economic capacity was associated with continued living independently of one’s children and with a greater probability of survival at 3 years. And a lower economic capacity was associated with a lower probability of improving functional capacity in a situation of prolonged stress, such as confinement due to the COVID-19 pandemic, a situation that ended up affecting the survival of these people. All this in a socioeconomically disadvantaged neighborhood, indicating, possibly, that small economic improvements can have great effects on survival (45, 46).

In this line, a low level of education, a lower economic capacity, and a higher level of dependence mark the future perspectives (47), and these factors, their impact, their prevalence, must be taken into consideration to develop strategies to modify their effect on the quality of life and survival of individuals (48, 49). And to reduce their inheritance.

Heredity may have multiple features that mask it. The pattern observed in this study shows a complex network. In this network, the family, as a care unit, has more nuances than are usually the object of study, which generally focus on the overload of the caregiver and the “burden” of the dependent (50). As nodes within this network that frames the family structure, and in relation to the children, one in five adult children did not manage to emancipate themselves and continued to live with their parents. These adult children, of a high mean age, have a potential risk of future social intervention since they coexist and show a clear interrelation between the situations of not having a job, not having their own home, and being single.

And two situations in relation to children were particularly relevant. On the one hand, the data obtained suggest the existence of a hidden population, that of sons who act as essential family caregivers. The socioeconomic situation of these sons makes visible a social reality, that of the generations that did not manage to get ahead. And this situation generates vulnerable populations, who are invisible because they are protected under the family umbrella. Evidencing an inverse dependency that frames an intergenerational inequality (51). A situation of frustrated independent life with a return to the parental home, which is shared by some of the women who are essential family caregivers. And whose cause lies in the socioeconomic difficulties that the population is currently through (52), which force other models of household coexistence (53–55). However, they also suggest that socioeconomically disadvantaged people are more likely to end up carrying out the essential family caregiver role, which can reinforce socioeconomic inequalities (56, 57).

In addition, and equally relevant, it is observed that, in Spanish culture and in socioeconomically disadvantaged environments (58), an archaic role in parental care is maintained. This archaic pattern shows that the role of essential family caregiver is mostly exercised by the eldest daughter of the family, who often does not work, does not have her own home, is not married, and is dedicated to the care of her parents (59). This situation generates intergenerational dependency. Although in this type of social environment this situation probably also occurs in households without ADL dependents, the need for care that dependency entails makes it more likely. Dependency thus becomes a factor that contributes to the maintenance of social and gender inequalities (60–63).

Independently of the cause that generates the cohabitation with the parents, the result for these children, mostly women (58), is a personal situation that transforms them *de facto* into socioeconomically dependent persons. People whose subsistence depends both on the economic income of the dependent person and on the habitational solution provided by that person. This reflects a precariousness that is transmitted from generation to generation and that leads these people to not progress in social status. This at-risk population, with a mean age that exceeds the pre-retirement age, may have problems accessing their inheritance when the ADL-dependent person dies, due to a lack of financial resources to pay for it. And this temporal dynamic has relevant implications for the social and healthcare systems, reflecting, in addition, multiple inequalities that must be addressed (64, 65).

However, there are more nuances. The data obtained also show a difference between the number of cohabiting children and the number of residents in these households, which hides another population that is usually invisible in the studies (66, 67). And that ostensibly increases the number of people under the family umbrella.

Finally, the deficiencies detected in this study with respect to the functionally dependent population should be taken into consideration by Social Services. ADL dependency is one of the main reasons for institutionalization (68). And the current approach to this socio-health problem differs to such an extent that it is difficult to maintain the principle of equity (45, 69, 70). The institutionalized dependent population receives protocolized care and daily follow-up, while non-institutionalized dependent patients form a group that is to a certain extent invisible and without this personalized care plan (71–73).

This study aimed from a holistic viewpoint to detect all the nuances that make up the network of care for people with functional dependency and ended up framing a model that provides relevant information to the social and healthcare systems (74, 75).

Among this information, the percentage of the variance in survival that explained the housing situation, the level of economic income, and the level of dependency itself indicates that many other factors are involved in the survival of the functionally dependent population, which will have to be established.

About data analysis, the Cox proportional hazards model is the most widely used multivariate model when analyzing a situation in two dimensions: time and event. Analyzing the possible limitations of this study, during the 3-year follow-up period there was a loss of patients due to transfer to another home, the causes of which were not recorded. For this reason, and although they were still alive at the end of the study, they were not included in the survival analysis using Cox regression. For the rest of the cohort, the housing situation did not change during the 3-year follow-up period.

In relation to the covariates included in the regression model, the level of economic income was updated for each economic level group in relation to the CPI. This updating allowed this variable to behave as a constant for the purposes of the study. However, because of home confinement due to the COVID-19 pandemic, the level of dependency did change. This result should be considered in the assessment of the results obtained.

The small sample size in the male group is also a limitation of this study. Although statistical tests were used to assess the comparability of the two groups, this fact should be considered in the comparison with other studies. This circumstance also occurs within the group “living with people other than adult children,” where a part of the dependent persons live with people who are indirect family (sister-in-law, brother-in-law) and another part with internal caregivers. On the other hand, this study revealed the existence of many people who are not adult children living in the homes of the dependent persons, but it was not designed to analyze this group. The influence of this group on the results observed is unknown.

Finally, some subsets of the data are small, so the results observed in their analysis will have to be confirmed by other studies.

## Conclusion

The population aging and the decline in the birth rate are altering the traditional balance that supported the model of care for the older persons. These factors are aggravated by the economic crisis, job precariousness, and the delay in the age of having the first child in the current generations. On the other hand, the baby-boom generation will contribute a considerable percentage of older adult people in the coming decades, altering the balance of the classic population pyramids. This situation will increase the number of people with functional dependency. All of this is within a context where generational changes in family size could cause a crisis of resources in the care of an increasingly aging population. Monitoring these changes becomes an unavoidable objective, since their consequences will affect not only the health field, but all levels of society.

This study is a first step along these lines. Through it, we have observed a model of care for the older adult in a socioeconomically disadvantaged environment. The results obtained show the importance of a holistic perspective in the assessment of functional dependence, rather than focusing solely on health factors, and have implications for socio-health planning.

Among dependent people, economic capacity influenced the ability to maintain an independent life and affected their survival at 3 years of follow-up. In women, not having a partner was a risk factor for losing independence and having to live with adult children. Having children implied a greater likelihood of being cared for as we advanced into old age, with an additional advantage when that child was a daughter. The provision of assistants for domestic tasks and personal care did not meet the dependency criteria, with the absence of family caregivers being one of the factors involved in this result. Along the same lines, the absence of assistance with personal care and household tasks in patients with severe functional dependence who live alone or with their older adult partner probably reflects social inequality, compared to people with similar characteristics who are institutionalized. Finally, loss of mobility associated with wheelchair dependence was a significant predictor of long-term mortality. The use of crutches as a complement to maintain mobility presented differences that advise specific study.

Above all, it provides relevant data on blind spots in the social coverage system. Pockets of poverty, intergenerational dependence, and a baseline situation that, foreseeably, will tend to worsen in the coming decades, generating not only that these generations do not maintain the social status of their parents, but that they lose it. All this in a social environment that, as a neighborhood, contributes to perpetuating social inequalities.

In addition to monitoring the impact of population aging on the model of care for the older adult, these results suggest that studies should also be carried out to address the social inequalities that may be generated in the environment of dependent persons. Studies are also needed to address their impact on socioeconomic indicators, both at the neighborhood level, to avoid the creation of ghettos, and at the municipality level, to establish measures of inter-territorial balance.

On the other hand, this study shows that intergenerational dependency has implications for social support for the older people, and that the current economic crisis may be contributing to it being a rising model. Socioeconomically disadvantaged people are more likely to end up performing the role of essential family caregiver. However, basing care for the older adult on the needs of others cannot be a socially acceptable model. These poverty traps only contribute to the deterioration of the social and economic structure of the areas where they are established, generating more poverty. Therefore, governments and institutions with responsibility for these problems should take appropriate measures to prevent the perpetuation of inequalities. Poverty should not be the good to be inherited.

Finally, the holistic perspective made it possible to observe patterns and networks, which activated/deactivated adaptive heuristics in the face of the diverse situations faced by the people who made up this community. This perspective could facilitate the development of models on which to base health planning. And, in any case, it allows us to understand why things end up being a certain way, allowing us to adapt our model of care to that reality.

## Data Availability

The original contributions presented in the study are included in the article/supplementary material, further inquiries can be directed to the corresponding author.
