# Erratum: The care of non-institutionalized ADL-dependent people in the Orcasitas neighborhood of Madrid (Spain) during the Covid-19 pandemic and its relationship with social inequalities, intergenerational dependency and survival

**DOI:** 10.3389/fpubh.2024.1513570

**Published:** 2024-11-11

**Authors:** Frontiers Production Office

**Affiliations:** Frontiers Media SA, Lausanne, Switzerland

**Keywords:** activities of daily living, social inequalities, intergenerational dependency, gender inequalities, essential family caregiver, COVID-19, wheelchair, functional impairment

Due to a production error, the **Conflict of Interest Statement** in the XML was incorrectly stated as:

“NS was employed by Senex. ES was employed by Frezent Biological Solutions.

The remaining authors declare that the research was conducted in the absence of any commercial or financial relationships that could be construed as a potential conflict of interest.”

This has been corrected to: “The authors declare that the research was conducted in the absence of any commercial or financial relationships that could be construed as a potential conflict of interest.”

The conflict of interest statement was correct in the PDF and HTML versions at time of publication. The publisher apologizes for this mistake.

The original version of this article has been updated.

